# Aging reveals a sex-dependent susceptibility of sarcospan-deficient mice to cardiometabolic disease

**DOI:** 10.1152/ajpheart.00702.2023

**Published:** 2024-08-09

**Authors:** Aida Rahimi Kahmini, Isela C. Valera, Rhiannon Q. Crawford, Luaye Samarah, Gisienne Reis, Salma Elsheikh, Rosemeire M. Kanashiro-Takeuchi, Nazanin Mohammadipoor, Bolade S. Olateju, Aaron R. Matthews, Michelle S. Parvatiyar

**Affiliations:** ^1^Department of Health, Nutrition, and Food Sciences, Florida State University, Tallahassee, Florida, United States; ^2^Department of Molecular and Cellular Pharmacology, University of Miami Miller School of Medicine, Miami, Florida, United States; ^3^Interdisciplinary Stem Cell Institute, University of Miami Miller School of Medicine, Miami, Florida, United States

**Keywords:** aging, cardiometabolic disease, inflammation, obesity, sex differences

## Abstract

Numerous genes including sarcospan (SSPN) have been designated as obesity-susceptibility genes by human genome-wide association studies. Variants in the *SSPN* locus have been linked with sex-dependent obesity-associated traits; however, this association has not been investigated in vivo. To delineate the role SSPN plays in regulating metabolism with potential to impact cardiac function, we subjected young and aged global SSPN-deficient (SSPN^−/−^) male and female mice to obesogenic conditions (60% fat diet). We hypothesized that loss of SSPN combined with metabolic stress would increase susceptibility of mice to cardiometabolic disease. Baseline and end-point assessments of several anthropometric parameters were performed including weight, glucose tolerance, and fat distribution of mice fed control (CD) and high-fat (HFD) diet. Doppler echocardiography was used to monitor cardiac function. White adipose and cardiac tissues were assessed for inflammation by histological, gene expression, and cytokine analysis. Overall, SSPN deficiency protected both sexes and ages from diet-induced obesity, with a greater effect in females. SSPN^−/−^ HFD mice gained less weight than wild-type (WT) cohorts, while SSPN^−/−^ CD groups increased weight. Furthermore, aged SSPN^−/−^ mice developed glucose intolerance regardless of diet. Echocardiography showed preserved systolic function for all groups; however, aged SSPN^−/−^ males exhibited significant increases in left ventricular mass (CD) and signs of diastolic dysfunction (HFD). Cytokine analysis revealed significantly increased IL-1α and IL-17Α in white adipose tissue from young SSPN^−/−^ male mice, which may be protective from diet-induced obesity. Overall, these studies suggest that several sex-dependent mechanisms influence the role SSPN plays in metabolic responses that become evident with age.

**NEW & NOTEWORTHY** Young and aged sarcospan (SSPN)-deficient mice were examined to assess the role of SSPN in obesity and cardiometabolic disease. Both sexes displayed a “leaner” phenotype in response to high-fat diet (HFD). Notably, several sex differences were identified in aged SSPN-deficient mice: *1*) females developed glucose intolerance (control and HFD) and *2*) males exhibited increased left ventricular mass (control) and diastolic dysfunction (HFD). Therefore, we conclude that SSPN exerts a sex-dependent influence on obesity-associated diseases.

## Graphical abstract

**Figure:**
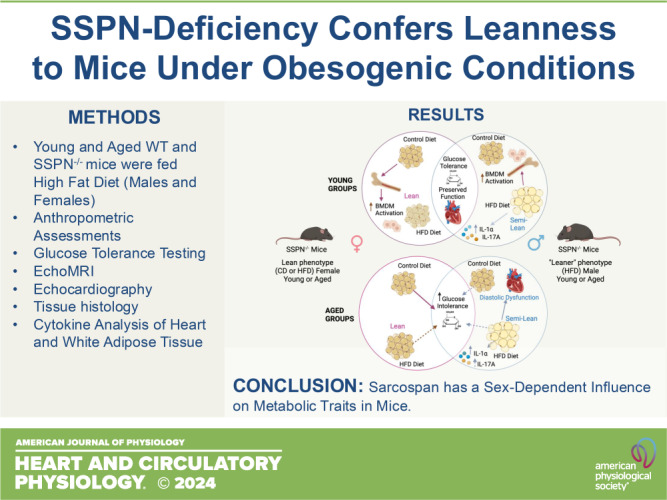

## INTRODUCTION

Obesity is on the rise throughout the world and contributes to the development of metabolic and cardiovascular diseases. A recent global report estimates that roughly 500 million adults are obese with a body mass index (BMI) of 30 or higher (1). Weight gain and obesity are accompanied by accumulation of abdominal fat, which heightens risk of cardiovascular disease and premature death. Adipose tissue releases a multitude of bioactive factors that influence adipocyte homeostasis and insulin resistance. Development of insulin resistance is associated with classical cardiovascular risk factors that include abnormalities in lipid storage, glucose intolerance, and hypertension. The heart is also impacted by insulin resistance, and decreased glucose uptake can undermine normal cardiac function (2). In addition, obesity predisposes individuals to developing cardiometabolic syndrome (CMS) (3). CMS is classified as a cluster of metabolic dysfunctions including insulin resistance, impaired glucose handling, dyslipidemia, hypertension, and central adiposity (4).

It has been established that obesity is highly heritable and influenced by a number of factors including gene interactions, environment, and behavior (5). The list of genes associated with obesity susceptibility is growing, and many of these gene associations remain to be verified. Several human genome-wide association studies (GWAS) have labeled the *SSPN* gene, an obesity susceptibility gene (6–12). *SSPN* encodes the protein sarcospan (SSPN), a small tetraspanin-like membrane protein that is part of the dystrophin-glycoprotein complex (DGC) and plays a role in maintaining muscle adhesion and sarcolemma stability (13–17). The SSPN protein is abundantly expressed in striated (18, 19) and smooth (20) muscle, and RNA expression has been detected in other tissues including adipose and female and male reproductive structures (21). The physiological role of the SSPN protein in striated muscle has been previously elucidated in SSPN-null (SSPN^−/−^) mice (17, 19, 22). The results of these studies prompted several preclinical studies that examined SSPN as a therapeutic agent to stabilize muscle membranes in Duchenne muscular dystrophy (DMD) mice (14–17). Pathogenic variants in DGC proteins have been demonstrated to cause various skeletal muscle, heart, and neurological conditions (23, 24). More recently, it has been shown that these DGC-related disease conditions also manifest with metabolic disturbances that affect insulin sensitivity and subsequent ectopic fat infiltration in muscle (25–30). Therefore, the connection of SSPN with metabolism is not unprecedented.

Additional GWAS studies have identified single-nucleotide polymorphisms (SNPs) within the *SSPN* locus that show strong sexual dimorphism in females (21, 31). The connection of *SSPN* with obesity, however, has not been verified in vivo. Earlier familial GWAS studies reported SNPs in the chromosome region 12p11 that segregates with specific traits including increased waist circumference (WC) and left ventricular (LV) mass (32). The human SSPN gene locus is located at 12p12.1 (NCBI Gene). Variants within the SSPN locus have been primarily associated with increased midsection adiposity or waist-to-hip ratio (WHR), WC, and WC/WHR (33), cardiometabolic disease traits including hypertension (34), and metabolic (10, 35) and cardiovascular (10, 32, 36–38) risk factors. The genetic association between SSPN expression and increased left ventricular mass may be a secondary effect of either altered metabolic state or increases in systolic, diastolic, and pulse blood pressures (34, 39). These studies suggest that SSPN has an important yet unknown contribution to key predisposing factors underlying development of cardiometabolic syndrome (CMS).

Although GWAS provides a tool to understand the complex genetics underlying increased susceptibility to obesity, it is unable to uncover biological mechanisms relative to specific genetic risks (40). Therefore, it is necessary to conduct experiments directed at uncovering the mechanisms underlying obesity-associated SNPs that have been identified in the *SSPN* gene promoter (21, 31, 41) and understand their contribution to cardiometabolic disease. The present study examines sex differences in white adipose tissue (WAT) distribution, glucose tolerance, white adipose and cardiac tissue inflammation, and cardiac function in mice lacking SSPN protein expression. Cardiovascular diseases present differently between sexes and therefore should be extensively investigated to identify sex-dependent therapeutic targets that can precisely address the issue (42). To better understand the role of SSPN throughout lifespan, we extended our studies of SSPN^−/−^ mice to understand the role of SSPN in aging-related phenomena that drive metabolism-related diseases.

## MATERIALS AND METHODS

### Mouse Models

Mice used in this study were wild-type (WT) (C57BL/6J) (JAX No. 000664) and SSPN-deficient (JAX No. 006837) mice obtained from Jackson Laboratories. The SSPN-deficient mice were developed by the Campbell laboratory (19) and donated to Jackson Laboratories. In our laboratory the SSPN-deficient (SSPN^−/−^) mice were backcrossed twice with C57BL/6J, and heterozygous crosses were used to obtain WT controls and SSPN^−/−^ mice. Group housing of mice of both sexes allowed mice to participate in normal behavior patterns, although at a cost to measuring individual food consumption. Group housing of young female mice stimulates and helps coordinate the estrus cycle of the mice. Aged female mice were also maintained in group housing.

### Study Design

In this study 16 groups of mice were evaluated. The young mice were assessed after a shorter diet course (4 mo), whereas the aged mice were assessed after a longer diet course (5 mo) to better study the impact on the cardiovascular system. The young mouse groups were 2–2.5 mo old at the initiation of a 4-mo high-fat (HFD) or control (CD) diet regimen. Therefore, at final analysis the mice in the young groups were ∼6–6.5 mo old. The total numbers of young mice included in the study were as follows: *1*) WT male CD (*n* = 21), *2*) WT female CD (*n* = 12), *3*) WT male HFD (*n* = 14), *4*) WT female HFD (*n* = 19), *5*) SSPN^−/−^ male CD (*n* = 23), *6*) SSPN^−/−^ female CD (*n* = 19), *7*) SSPN^−/−^ male HFD (*n* = 11), and *8*) SSPN^−/−^ female HFD (*n* = 13). The aged/old mouse groups were ∼12 mo old at the initiation of the 5-mo HFD and CD regimen. Therefore, the “aged” groups of mice in this study were ∼17 mo old at final analysis. The total numbers of aged mice included in this study were as follows: *9*) WT male CD (*n* = 20), *10*) WT female CD (*n* = 12), *11*) WT male HFD (*n* = 9), *12*) WT female HFD (*n* = 6), *13*) SSPN^−/−^ male CD (*n* = 24), *14*) SSPN^−/−^ female CD (*n* = 18), *15*) SSPN^−/−^ male HFD (*n* = 8), and *16*) SSPN^−/−^ female HFD (*n* = 10).

### High-Fat Diet Administration

Both male and female WT and SSPN-deficient mice were fed Envigo high-fat diet (TD.06414) (60% fat content, 5.1 kcal/g). The approximate fatty acid profile (60% of total fat) consisted of 36% saturated, 41% monosaturated, and 23% polyunsaturated fat. Initial data (not shown) were obtained using the Envigo control diet (Cat. No. TD.08806; 10% fat content, 3.6 kcal/g) as recommended by the manufacturer; however, it was found incompatible for the SSPN^−/−^ mice, which exhibited liver and kidney enlargement after 4 mo on the diet. Instead, the standard chow diet (CD) (LabDiet, Cat. No. 5001-RHI-E 14) (4.5% crude fat) was fed to the mice ad libitum. The duration of the diet regimen for the young mice was 4 mo starting at 2 mo of age, and for the old mice the diet was extended to 5 mo starting at 12 mo of age.

### Anthropometric Analysis

The mice in this study were fed either normal chow diet (CD) or HFD and were weighed once a month to assess changes in weight. In addition, food intake was monitored and weighed. After 4 mo of diet(s) the young mice were weighed, and the aged mice were weighed after 5 mo of diet(s). After euthanasia, tissues were collected and the weights of heart, liver, and visceral fat pads and the tibia length were reported.

### Tissue Histology

After harvest the tissues were embedded in OCT, immediately flash frozen in liquid N_2_-cooled 2-methylbutane (Sigma-Aldrich, VWR Cat. No. SIAL277258-1L), and stored at −80°C until further use. The tissues were next sectioned and cut to 7-µm thickness.

Hematoxylin and eosin (H&E) staining was used to visualize changes in tissue architecture and the occurrence of fibrosis as previously described (17). For wet mount imaging of the white adipose tissue, the tissues were first fixed in a 4% paraformaldehyde (PFA) solution before imaging. Wet mount images were used to measure adipocyte diameters. The images were captured under identical conditions with a Leica DMI4000B inverted microscope using ×10 and ×20 objectives and image acquisition Axiovision Rel 4.5 software.

### Glucose Tolerance Testing

Pre- and postdiet glucose tolerance tests were conducted for all mice in the study. After mice were fasted overnight (∼15 h), they were injected with an intraperitoneal bolus of 1 g/kg d-(+)-glucose (Cat. No. G021, Sigma-Aldrich), administered first thing in the morning. The blood samples were obtained from the mice using the tail-nick method, and blood sugar levels were monitored with a glucometer (Cat. No. 08396-5001-75, Advanced Glucose Meter). Blood glucose readings were assessed at 0, 15, 60, 120, and 180 min time points for males and 0, 15, 60, and 120 min time points for females.

### EchoMRI Imaging

Body composition measurements were taken for mice at the beginning and completion of the study with an EchoMRI-100H system (http://www.echomri.com/). Measurements were taken of conscious mice, and sunflower oil was used as a 100% fat standard to calibrate the system before data acquisition. Mice were weighed before measurements, and data generated were percent fat, percent lean, and percent water based upon weight of the mouse.

### Tissue Cytokine Analysis

Several approaches were used to access tissue cytokine levels. The proteome profiler antibody array, mouse XL cytokine array kit (Cat. No. ARY028, R&D Systems), was first used in young male mice to screen tissues for altered cytokines according to manufacturer’s directions. For the WAT and heart samples, 40 µg of tissue was used. The remaining mouse groups were analyzed for changes in selected cytokines with the Luminex discovery assay mouse premixed multi-analyte kit (Cat. No. LXSAMSM, R&D Systems) according to manufacturer’s recommendations. The tissue was lysed in PBS, 1% protease inhibitor, and 0.2% Triton X-100 buffer composition. The cytokine array data were analyzed with MyAssays software (MyAssays, 2009) and reported as relative expression values.

### Quantitative Real-Time Polymerase Chain Reaction

To assess alterations in gene expression, total RNA was isolated from heart tissue with Invitrogen TRIzol Reagent (Cat. No. 15-596-018) and purified according to manufacturer’s recommended procedures. RNA was quantified with the Nanodrop One (Cat. No. 840274100, Thermo Scientific). cDNA synthesis was performed with the iScript cDNA synthesis kit (Cat. No. 1708891, Bio-Rad) with 0.5 μg of total RNA per reaction according to manufacturer’s recommendations. Quantitative real-time polymerase chain reaction (qRT-PCR) was performed to analyze gene-specific expression with a QuantStudio 3 PCR machine (Applied BIOsystems). Melt curves were performed to detect the presence of primer dimers. Gene-specific expression was assessed using primers (Integrated DNA Technologies, IDT) designed to detect atrial natriuretic peptide (ANP), and sarcospan transcripts with β-actin were used as the housekeeping gene. For cytokines the following primers were used to detect IL-6, TNFα, and IL-1β transcripts, with L32 used as the housekeeping gene. Changes in relative gene expression were quantified with the 2^−ΔΔCT^ method. The following primers were used:

ANP: forward, 5′-GCT TCC AGG CCA TAT TGG GAG, reverse, 5′-GGG GGC ATG ACC TCA TCT T

SSPN: forward, 5′-TGC TAG TCA GAG ATA CTC CGT TC, reverse, 5′-GTC CTC TCG TCA ACT TGG GTA TG

β-Actin: forward, 5′-GGC TGT ATT CCC CTC CAT CG, reverse, 5′-CCA GTT AAC AAT GCC ATG T

IL-6: forward, 5′-ACA ACC ACG GCC TTC CCT ACT T, reverse, 5′-CAC GAT TTC CCA GAG AAC ATG TG

TNFα: forward, 5′-GAC GCG GAA CTG GCA GAA GAG, reverse, 5′-TTG GTG GTT TGT GAG TGT GAG

IL-1β: forward, 5′-GCA ACT GTT CCT GAA CTC AAC T, reverse, 5′-ATC TTT TGG GGT CCG TCA ACT

L32: forward, 5′-TTA AGC GAA ACT GGC GGA AAC, reverse, 5′-TTG TTG CTC CCA TAA CCG ATG

### Immunoblotting

Tissues were collected and ground in liquid nitrogen using a mortar and pestle. The ground tissue was solubilized in RIPA buffer containing Pierce Protease Inhibitor (Cat. No. A32955, Thermo Scientific), kept on ice, and homogenized with a Dounce homogenizer. After homogenization, the heart, skeletal muscle (quadriceps), and white adipose tissue (WAT) samples were spun at 12,000 rpm for 5 min at 4°C to remove insoluble proteins. Protein concentrations were quantified in triplicate with the 660-nm Protein Assay Reagent (Cat. No. 22660, Pierce). Sodium dodecyl sulfate-polyacrylamide gel electrophoresis (SDS-PAGE) 12% gels were loaded with 35 µg of protein diluted in Laemmli sample buffer and run at 100 V until the dye front reached the bottom. Proteins were transferred onto 0.2-µm nitrocellulose membranes (Cat. No. 1620112, Bio-Rad) at 300 mA for 1.5 h. Membranes were blocked while rocking in 3% bovine serum albumin (BSA) (Cat. No. 97061-420, VWR International) in Tris-buffered saline (TBS) containing 0.1% Tween 20 (TBST) for 1 h. The membranes were incubated overnight on a rocker at 4°C with primary antibodies diluted in 3% BSA including the anti-SSPN antibody (Cat. No. sc-393187, 1:500 dilution, Santa Cruz Biotechnology), and anti-β-actin antibody (Cat. No. 81115-1-RR, 1:1,000 dilution, Proteintech) was used as the loading control. The membranes were washed three times at 10 min each and then incubated while rocking with the respective secondary antibodies goat anti-mouse Ig-HRP (Cat. No. 1010-05, 1:5,000, Southern Biotech for SSPN) and goat anti-rabbit Ig-HRP (Cat. No. 4010-05, 1:10,000, Southern Biotech for Actin) diluted in 5% milk for 2 h at room temperature. Membranes were washed three times with TBST for 10 min each, incubated in Clarity Western ECL substrate (Cat. No. 1705061, Bio-Rad) for 5 min on a rocker, and imaged with a Bio-Rad ChemiDoc Imaging System.

### DNA Methylation Assay

Visceral white adipose tissue (WAT) was collected from mice and flash frozen in liquid N_2_ until further use. The WAT was weighed before use, and 50 mg of tissue was used for each sample. DNA was extracted with the Quick-DNA Miniprep Plus Kit (Cat. No. D4068, Zymo Research) according to manufacturer’s instructions. DNA methylation was assessed with the 5-mC DNA ELISA Kit (Cat. No. D5325-A, Zymo Research) following the instructions. A methylation standard was included, and any samples below this value were considered nonmethylated.

### Cardiomyocyte Size Determination

Wheat germ agglutinin (WGA) was used to stain cardiomyocyte borders of transverse cut heart sections obtained from mice in this study. Heart tissues were fixed in 4% paraformaldehyde (PFA) for 10 min, washed 3 × 5 min each in PBS + 0.1% Tween (PBST), and incubated in WGA (Fluor555-WGA conjugate, Cat. No. 25539, AAT Bioquest) at a final concentration of 10 µg/mL for 1 h. After staining, heart sections were washed three times in PBST for 5 min each. Tissues were mounted in ProLong Gold antifade reagent (Cat. No. P36934, Invitrogen) and covered with a coverslip. Images were taken using the RHOD filter cube, and ×10 images were stitched using a Leica DMi8 microscope to visualize the entire heart section. ImageJ was used to measure individual cardiomyocyte areas (∼100 cells per heart) in the left ventricular free wall of the heart.

### Echocardiography

Measurements were recorded of left ventricular (LV) size, wall thickness, mass, and ventricular and valve function, and Doppler blood flows were obtained by previously described methods (43–45). Briefly, mice were anesthetized with 1%–2% isoflurane, and body temperatures were maintained at 37°C. Hearts were imaged using the parasternal long-axis and short-axis views to determine cardiac morphology and function. Evaluation of diastolic function was performed using the ratio of the LV transmitral early peak flow (E wave) to late peak flow (A wave) velocity (*E/A* ratio) and isovolumic relaxation time measurements (IVRT; in ms). All echocardiographic assessments were analyzed offline with Vevo LAB 2.1.0 software, and Visual Sonics images were taken over three to five consecutive heartbeats.

### Statistics

All values in the text and figures are presented as means ± SE, unless otherwise indicated. Statistical significance was determined by using a two-tailed Student’s *t* test to compare two relevant groups that exhibited normality. Ordinary one-way ANOVA was used when comparisons were made across all groups, followed by Tukey’s multiple comparisons test. *P* values of <0.05 were considered significant.

### Study Approval

The animal studies included in this study were reviewed and approved by the Institutional Animal Care and Use Committee at Florida State University (Protocol No. IPROTO202200000003).

## RESULTS

### Assessment of Weight Changes in Young and Aged SSPN^−/−^ Mice in Response to High-Fat Diet

To determine whether SSPN influences the body weight of young (2 mo) and adult (12–13 mo) male and female mice, a high-fat diet study was initiated. *SSPN* gene expression has been documented in tissues throughout the body, and before this study we used quantitative real-time polymerase chain reaction (qRT-PCR) to examine its expression in WT, SSPN^+/−^, and SSPN^−/−^ hearts and white adipose tissue (WAT) from male mice and immunoblotting to evaluate protein levels in skeletal, cardiac and WAT (Supplemental Fig. S1; all Supplemental Figures are available at https://doi.org/10.6084/m9.figshare.26048563.v2). WT and global SSPN-deficient (SSPN^−/−^) mice were fed control (CD; 4.5% fat) or high-fat (HFD; 60% fat) diet for 4- and 5-mo periods, respectively. At the end of the study young mice were ∼6 mo of age (equivalent to a 20- to 30-yr-old human), whereas adult mice were ∼17–18 mo of age. According to Jackson Laboratories guidelines, an 18-mo-old mouse is equivalent to a 56-yr-old human and considered “old.” For the purposes of this study, this group is considered “aged” since they matched these criteria at the conclusion of this study. The diet studies were of different lengths, with the shorter diet regimen (4 mo) being administered to the young mouse groups and the aged mice subjected to a longer diet regimen (5 mo).

**Figure 1.**
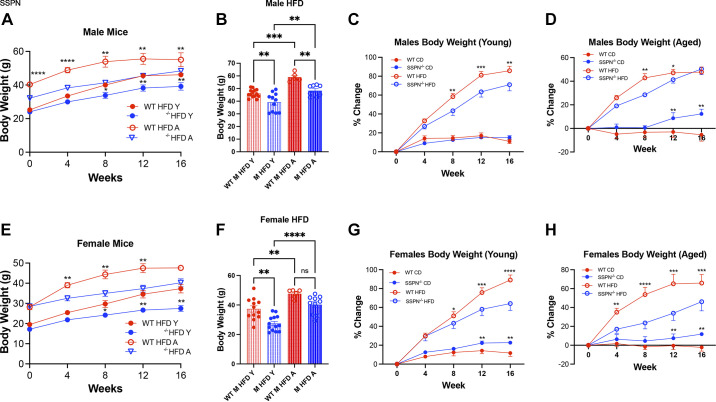
Sarcospan (SSPN) deficiency is protective against diet-induced obesity. *A*: results for male mice: change in body weight (BW, in g) over 16-wk high-fat diet (HFD) or control diet (CD) for wild-type (WT) and SSPN-deficient (SSPN^−/−^) male mice with young (Y) and aged (A) mice. *B*: terminal body weights of male mice after 16-wk HFD. *C* and *D*: percent change in BW for males in young (*C*) and aged (*D*) mice (*E*) results for female mice: change in BW (in g) over 16-wk HFD for WT and SSPN^−/−^ young and aged female mice. *F*: terminal BWs of female mice after 16-wk HFD. *G* and *H*: percent change in BW for females in young (*G*) and aged (*H*) mice. Data in *A*, *C–E*, *G*, and *H* show means ± SE. Data in *B* and *F* show individual values, and bar heights represent average values. Data were analyzed by 1-way ANOVA followed by Tukey’s post hoc analysis for young males [CD WT (*n* = 6) and SSPN^−/−^ (*n* = 6); HFD WT (*n* = 14) and SSPN^−/−^ (*n* = 11)], young females [CD WT (*n* = 6) and SSPN^−/−^ (*n* = 5); HFD WT (*n* = 12) and SSPN^−/−^ (*n* = 13)], aged males [CD WT (*n* = 6) and SSPN^−/−^(*n* = 7); HFD WT (*n* = 6) and SSPN^−/−^ (*n* = 8)], and aged females [CD WT (*n* = 5) and SSPN^−/−^ (*n* = 5); HFD WT (*n* = 6) and SSPN^−/−^ (*n* = 10)]. Significant values: *P < 0.05, **P < 0.01, ***P < 0.001, and ****P < 0.0001. ns, Not significant.

In Fig. 1, body weight (BW) measurements are shown for the different groups of mice in response to HFD. The time course of weight changes is presented in Fig. 1*A* for male mice and in Fig. 1*E* for female mice. The overall trend in the young mouse groups was that both male and female SSPN^−/−^ mice gained significantly less weight than WT mice during the HFD course (Fig. 1, *A* and *E*). Overall, the different groups of mice had similar weights at baseline. However, there were two exceptions, with young SSPN^−/−^ female mice weighing less at baseline than matching WT controls and aged male SSPN^−/−^ mice weighing less than their WT counterparts (Fig. 1, *A* and *E*). Since these two groups of SSPN^−/−^ mice weighed significantly less at baseline, comparing the percent BW change during the diet period may be more informative (Fig. 1, *C*, *D*, *G*, and *H*). The final weights of the mice in this study were directly compared for young and aged male mice (Fig. 1*B*) and for young and aged female mice (Fig. 1*F*). Upon completion of the diet studies the final body weights (in g) were compared, and both young male and female SSPN^−/−^ mice gained significantly less weight than their respective WT controls (Fig. 1, *B* and *F*). After aging, the final weight was significantly lower in the SSPN^−/−^ male group only (see Fig. 1*B*).

After completion of the HFD study young WT male mice had a significantly higher change in average weight from their starting values, whereas HFD young SSPN^−/−^ male mice had a lower increase in average weight (Fig. 1*C*). In the aged mouse groups, male mice had lower percent change (Fig. 1*D*) in average weight gain in response to HFD compared with the young male groups (Fig. 1*C*). With aging, SSPN deletion in males, however, offered less protection from diet-induced obesity, with a similar percent body weight change for WT and SSPN^−/−^ mice, respectively (Fig. 1*D*). The percent body weight change in aged CD-fed males showed a different trend, with WT mice exhibiting an age-related decrease in BW or negative percent body weight change while the SSPN^−/−^ mice had a significant increase (Fig. 1*D*).

**Figure 2.**
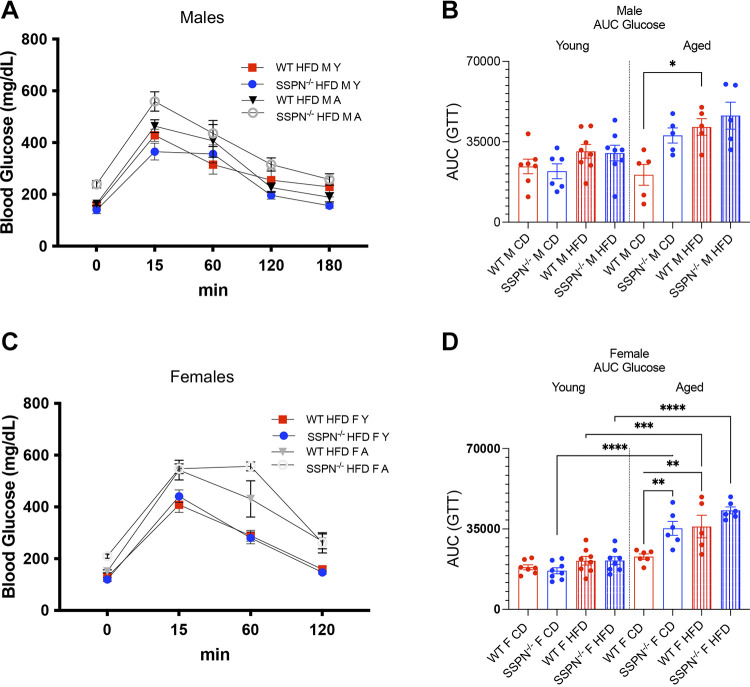
Aging uncovers glucose intolerance in sarcospan (SSPN)-deficient (SSPN^−/−^) mice. To assess glucose handling in young and aged mice, they were subjected to glucose tolerance testing (GTT). *A*: blood glucose values plotted as a function of time (0–180 min) for young (Y) and aged male HFD wild-type (WT) and SSPN^−/−^ mice after administration of a glucose bolus at *time 0*. HFD, high-fat diet. *B*: individual area under the curve (AUC) values depicting glucose clearance in young and aged male control diet (CD) and HFD mice after administration of a glucose bolus. *C*: blood glucose values plotted as a function of time (0–120 min) for young and aged female HFD WT and SSPN^−/−^ mice after glucose bolus administration from *time 0*. *D*: individual AUC values for female CD and HFD mice after glucose bolus administration. Data are plotted as averages in *A* and *C* and as individual values in *B* and *D*; bar height indicates the average value. Errors are shown as means ± SE. Data were analyzed by 1-way ANOVA followed by Tukey’s post hoc analysis, and numbers of mice in groups are included in Table 1 and Table 2. Significant values: *P < 0.05, **P < 0.01, ***P < 0.001, and ****P < 0.0001.

Young SSPN^−/−^ female mice had significantly lower BW than WT females after completion of the HFD study. However, they had lower initial baseline weights that can be observed in Fig. 1*E*. The percent body weight change was compared between the young female mice in response to HFD, and WT mice had a significant increase in body weight compared with SSPN^−/−^ mice (Fig. 1*G*). In the aged female HFD group, the SSPN^−/−^ mice had a significantly lower percent body weight change compared with WT mice (Fig. 1*H*). During the diet study, SSPN^−/−^ mice fed control diet had significantly higher percent body weight change compared with WT controls, except for the young male mouse group as in Fig. 1*C*. This suggests that SSPN deficiency alters additional parameters independent of diet-induced obesity. Food consumption was not significantly different between the HFD young mouse groups; however, aged SSPN^−/−^ HFD mice appeared to be consuming more kilocalories per day as seen in Supplemental Fig. S2, though their weights remained lower than their respective WT controls.

### Determining the Impact of SSPN Deletion on Glucose Tolerance in Mice

To assess the impact of SSPN deletion on glucose tolerance, mice were fasted overnight and subjected to glucose tolerance testing. The glucose response curves are shown for male mice of all HFD groups in this study in Fig. 2*A* and Table 1. Fasting baseline blood glucose levels were significantly increased in several groups including the aged CD and HFD SSPN^−/−^ female mice and HFD SSPN^−/−^ male mice, which were above 200 mg/dL and considered in the diabetic range. Baseline fasting glucose levels were higher than expected, and for the SSPN^−/−^ HFD groups several mice had blood glucose levels above the 600 mg/dL cutoff for the glucometer, meaning that the average values are slightly higher than reported.

**Figure 3.**
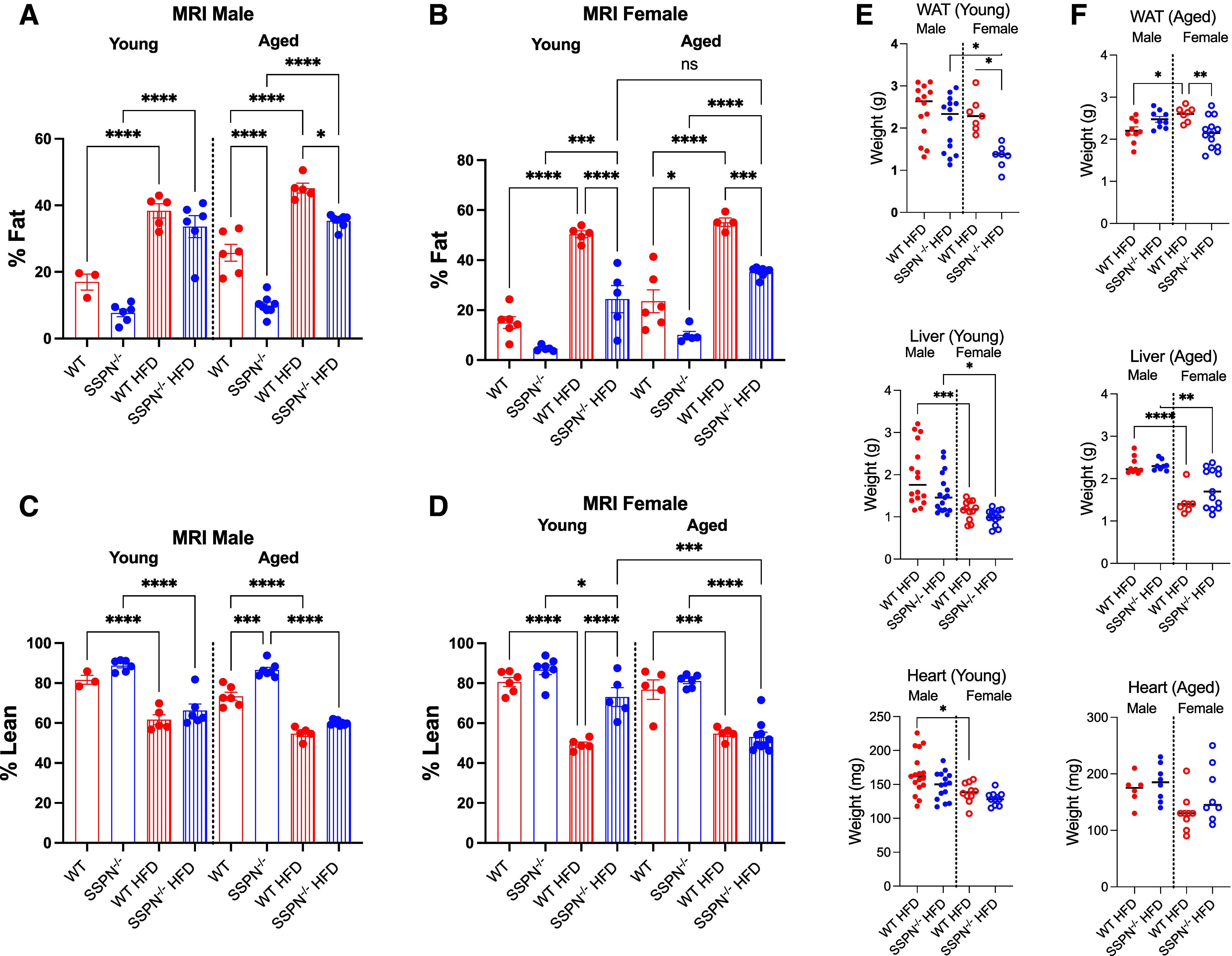
Sarcospan (SSPN) deficiency causes distinct alterations in white adipose tissue (WAT) distribution after high-fat diet (HFD). EchoMRI was used to assess the whole body composition of mice after 12 wk of either control diet (CD) or HFD. *A*: percent fat values from male young and aged wild-type (WT) and SSPN-deficient (SSPN^−/−^) mice. *B*: percent fat measurements from female young and aged WT and SSPN^−/−^ mice. *C*: percent lean measurements from male young and aged WT and SSPN^−/−^ mice. *D*: percent lean measurements from female young and aged WT and SSPN^−/−^ mice. *E* and *F*: weights of visceral WAT, liver, and heart after CD or HFD for young male and female (*E*) and aged male and female (*F*) mice. Data were analyzed by 1-way ANOVA followed by Tukey’s post hoc analysis. Numbers of mice used in this study are included in Table 3 and Table 4. Significant values: *P < 0.05, **P < 0.01, ***P < 0.001, and ****P < 0.0001.

**Table 1.** Summary of glucose tolerance testing in control and HFD young and aged male mice

	Young				Aged			
	WT CD	SSPN^−/−^ CD	WT HFD	SSPN^−/−^ HFD	WT CD	SSPN^−/−^ CD	WT HFD	SSPN^−/−^ HFD
*n*	6	6	8	8	5	5	5	5
	*Average blood glucose levels, mg/dL*							
*Time*, min								
* 0*	187.2 ± 14.8	175.5 ± 9.7	156.7 ± 6.3	139.7 ± 14.2	141 ± 5.8	146.6 ± 14.3	163.6 ± 6.8	239.2 ± 14.1^a,b,c^
* 15*	350.2 ± 36.9	328.4 ± 29.8	428.3 ± 23.6	365.2 ± 32.3	398.6 ± 43.4	409.6 ± 57.4	463.6 ± 24.9	559 ± 37.4^a^
* 60*	246.3 ± 20.2	213.2 ± 12.2	314.9 ± 36.3	356.4 ± 34.7	222.6 ± 41.3	382.8 ± 72.6	406.8 ± 62.9	435.4 ± 50.4
* 120*	207.2 ± 21.7	217.4 ± 19.7	255.2 ± 42.6	196.3 ± 14.9	150.8 ± 25.4	274.6 ± 44.3	226.4 ± 18.2	315.4 ± 25.4
* 180*	166.5 ± 12.4	159 ± 10.2	228.7 ± 38.5	156.2 ± 9.3	116 ± 26.1	172.8 ± 19.2	189 ± 18	257.6 ± 22.2

Values are means ± SE; *n*, number of mice. CD, control diet; HFD, high-fat diet; SSPN^−/−^, sarcospan deficient; WT, wild type. One-way ANOVA was used to compare differences between groups. ^a^*P* < 0.05, SSPN^−/−^ (young vs. aged) HFD mice; ^b,^^c^*P* < 0.05, SSPN^−/−^ CD vs. HFD (aged) mice. Significant groups: *Time 0 min*: ****P < 0.00001, SSPN^−/−^ HFD male (young vs. aged); *Time 15 min*: **P < 0.00001, SSPN^−/−^ HFD male (young vs. aged), SSPN^−/−^ CD male.

The aged SSPN^−/−^ male mice had the overall highest acute spike in blood glucose at 15 min compared with aged WT male mice (Fig. 2*A* and Table 1). The areas under the curve (AUCs) are shown for the male mouse groups in Fig. 2*B*, and the only significant differences were seen in aged WT HFD mice compared with controls. With age, the male CD SSPN^−/−^ mice showed a larger AUC compared with CD WT mice, though the trend was not significant. The glucose tolerance of female mouse groups was compared, and the glucose response curve is shown in Fig. 2*C* and Table 2 and the AUC in Fig. 2*D*. Overall, the aged HFD female SSPN^−/−^ mice took longer to clear blood glucose, and it remained elevated for 60 min on average and peaked at 556.7 mg/dL. Young female mice showed a trend similar to male mice; however, the AUC values were slightly lower (Fig. 2*D*). With aging, however, significant differences emerged between the groups. The AUC values of aged female CD SSPN^−/−^ mice were significantly increased compared with young CD SSPN^−/−^ mice, indicating that aging affected their glucose tolerance (Fig. 2*D*). This, however, was not observed for the aged female CD WT mice. Aged female WT mice showed glucose intolerance only after the HFD course, as seen by the significant increase in AUC compared with aged CD WT females and young WT females fed HFD (Fig. 2*D*).

**Table 2.** Summary of glucose tolerance testing in control and HFD young and aged female mice

	Young				Aged			
	WT CD	SSPN^−/−^ CD	WT HFD	SSPN^−/−^ HFD	WT CD	SSPN^−/−^ CD	WT HFD	SSPN^−/−^ HFD
*n*	7	8	9	6	6	6	5	6
	*Average blood glucose levels, mg/dL*							
*Time*, min								
* 0*	151.7 ± 6.8	131.5 ± 8.9	129.7 ± 5.2	119.7 ± 7.9	143.5 ± 4.3^a^	226.2 ± 9.9^b^	151.2 ± 5.6	209.9 ± 8.3^d,f^
* 15*	363.9 ± 53.2	399.7 ± 45.1	408.7 ± 29.9	441.3 ± 24.1	547.2 ± 33.7	472.2 ± 41.9^e^	542.4 ± 38.1	547.7 ± 18.6
* 60*	205.2 ± 14.3	191.9 ± 17.2	288.9 ± 20.9	280.5 ± 23.1	231.9 ± 11	457.7 ± 42.6^b,e^	430.8 ± 70.1^c,g^	556.7 ± 17.2^d^
* 120*	175 ± 15.2	179.7 ± 12.8	158.9 ± 10.4	146.9 ± 6.5	175.7 ± 10	245.9 ± 8.9	267 ± 28.5^c,g^	261.5 ± 38.9^d^

Values are means ± SE; *n*, number of mice. CD, control diet; HFD, high-fat diet; SSPN^−/−^, sarcospan deficient; WT, wild type. One-way ANOVA was used to compare differences between groups. ^a^*P* < 0.05, WT (young vs. aged) CD mice; ^b^*P* < 0.05, SSPN^−/−^ (young vs. aged) CD mice; ^c^*P* < 0.05, WT (young vs. aged) HFD mice; ^d^*P* < 0.05, SSPN^−/−^ (young vs. aged) HFD mice; ^e^*P* < 0.05, WT vs. SSPN^−/−^ (aged) CD mice; ^f^*P* < 0.05, WT vs. SSPN^−/−^ (aged) HFD mice; ^g^*P* < 0.05, WT CD vs. HFD (aged) mice. Significant groups: *Time 0*: ****CD SSPN^−/−^ (young vs. aged), ****HFD SSPN^−/−^ (aged), *** CD WT vs. SSPN^−/−^ (aged), HFD WT vs. SSPN^−/−^; *Time 15*: *CD WT (young vs. aged; *Time 60*: ****CD WT vs. KO (aged), **WT CD vs. HFD (aged); and *Time 120*, **HFD WT (young vs. aged), ***HFD SSPN^−/−^ (young vs. aged), *WT CD vs. HFD (aged).

### Body Composition Measurements to Assess the Impact of Sarcospan on Adipose Distribution

Body composition measurements were obtained using EchoMRI to assess whether SSPN deletion affects adipose tissue distribution and percent body mass. The EchoMRI data were compiled for male mice (Table 3) and female mice (Table 4). When the CD groups of both sexes and ages were compared, it could be observed that SSPN^−/−^ mice had lower percent fat compared with respective WT controls; however, the trend was significant only in aged male SSPN^−/−^ mice (Fig. 3, *A* and *B*). In the HFD groups, only the aged male SSPN^−/−^ mice had significantly lower percent fat compared with the respective HFD WT control (Fig. 3*A*). Baseline differences, however, may be a contributing factor (Fig. 3*A*). Overall, the percent lean values were lowest in the mice with the greatest percent fat increase (Fig. 3, *C* and *D*). The weights of tissues collected after HFD are shown for young mice in Fig. 3*E* and for aged mice in Fig. 3*F*. In males, the visceral white adipose tissue (WAT), liver, and heart weights were largely unchanged between WT and SSPN^−/−^ mice at either age and are plotted in Fig. 3, *E* and *F*.

**Figure 4.**
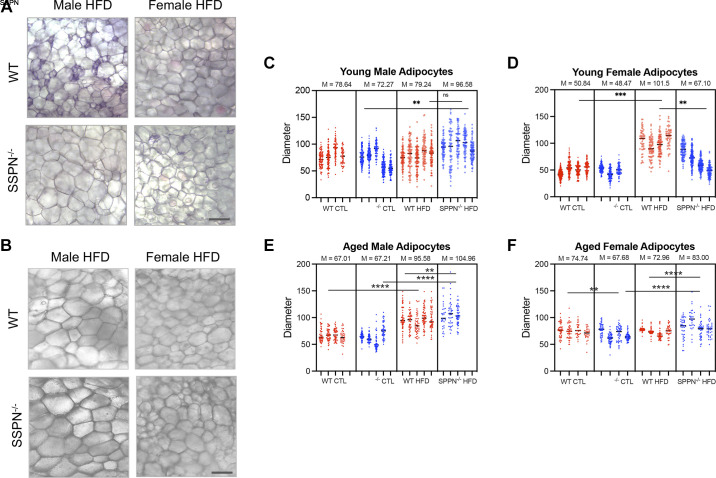
Adipocyte size is altered in some sarcospan (SSPN)-deficient (SSPN^−/−^) mouse groups after high-fat diet (HFD). *A* and *B*: wet mount images of visceral white adipose tissue (VAT) from wild-type (WT) and SSPN^−/−^ mice for young male and female HFD (*A*) and aged male and female HFD (*B*) mice. Scale bar indicates 50 μm. Measurements of adipocyte area were obtained as (pixels)^2^ using ×10 wet mount white adipose tissue images. *C–F*: adipocyte diameters were calculated from area measurements using *d* = 2*r* (in mm) for young males (*C*), young females (*D*), aged males (*E*), and aged females (*F*). Adipocyte diameters are shown for individual mice (*n* = 3–5), and mean values are reported above the graphs in *C–F*. CTL, control diet. Data were analyzed by 1-way ANOVA followed by Tukey’s post hoc analysis. Numbers of mice used in this study are included in Table 3 and Table 4. Significant values: **P < 0.01, ***P < 0.001, and ****P < 0.0001. ns, Not significant.

**Table 3.** Summary of body composition data in control and HFD young and aged male mice

	Young				Aged			
	WT CD	SSPN^−/−^ CD	WT HFD	SSPN^−/−^ HFD	WT CD	SSPN^−/−^ CD	WT HFD	SSPN^−/−^ HFD
*n*	3	6	5	6	6	7	5	8
	*EchoMRI values (males)*							
%Total fat	17 ± 2.4	7.7 ± 1.2	38.4 ± 2.2^a^	33.6 ± 3.3^b^	25.7 ± 2.5	10.1 ± 1.2^c^	45.2 ± 1.5^d^	35.3 ± 0.7^e,f^
%Total lean	81.7 ± 2.2	88.7 ± 1.2	61.7 ± 2.5^a^	66.3 ± 3.3^b^	73.4 ± 2.0	83.7 ± 1.3^c^	54.8 ± 1.4^d^	60.0 ± 0.4^e^

Values are means ± SE; *n*, number of mice. CD, control diet; HFD, high-fat diet; SSPN^−/−^, sarcospan deficient; WT, wild type. One-way ANOVA was used to compare differences between groups. ^a^*P* < 0.05, WT CD vs. HFD (young) mice; ^b^*P* < 0.05, SSPN^−/−^ CD vs. HFD (young) mice; ^c^*P* < 0.05, WT vs. SSPN^−/−^ (aged) CD mice; ^d^*P* < 0.05, WT CD vs. HFD (aged) mice; ^e^*P* < 0.05, SSPN^−/−^ CD vs. HFD (aged) mice; ^f^*P* < 0.05, WT vs. SSPN^−/−^ HFD (aged) mice. Significant groups: Fat (%): ****WT CD vs. HFD (young), ****SSPN^−/−^ CD vs. HFD (young), ****CD WT vs. SSPN^−/−^ (aged), ****WT CD vs. HFD (aged), ****vs. SSPN^−/−^ CD vs. HFD (aged), *WT HFD vs. SSPN^−/−^ HFD (aged); Lean (%): ****WT CD vs. HFD (young), ****SSPN^−/−^ CD vs. HFD (young), **CD WT vs. SSPN^−/−^ (aged), ****WT CD vs. HFD (aged), vs. ****SSPN^−/−^ CD vs. HFD (aged).

**Table 4.** Summary of body composition data in control and HFD young and aged female mice

	Young				Aged			
	WT CD	SSPN^−/−^ CD	WT HFD	SSPN^−/−^ HFD	WT CD	SSPN^−/−^ CD	WT HFD	SSPN^−/−^ HFD
*n*	6	7	5	5	6	4	4	10
	*EchoMRI values (females)*							
%Total fat	15.1 ± 2.4	4.6 ± 0.5	50.4 ± 1.4^a^	24.4 ± 5.4^b^	23.5 ± 4.6	10.7 ± 1.6^g^	55.2 ± 1.7^d^	43.0 ± 3.0^c,e,f^
%Total lean	80.6 ± 2.2	86.7 ± 2.4	49.3 ± 1.3^a^	73.1 ± 4.7^b^	75.4 ± 4.2	81.6 ± 1.7^g^	45.2 ± 1.1^d^	53.1 ± 2.4^c,e,f^

Values are means ± SE; *n*, number of mice. CD, control diet; HFD, high-fat diet; SSPN^−/−^, sarcospan deficient; WT, wild type. One-way ANOVA was used to compare differences between groups. ^a^*P* < 0.05, WT CD vs. HFD (young) mice; ^b^*P* < 0.05, SSPN^−/−^ CD vs. HFD (young) mice; ^c^*P* < 0.05, SSPN^−/−^ HFD (young vs. aged) mice; ^d^*P* < 0.05, WT CD vs. HFD (aged) mice, ^e^*P* < 0.05, SSPN^−/−^ CD vs. HFD (aged) mice; ^f^*P* < 0.05, WT HFD vs. SSPN^−/−^ HFD (aged) mice; ^g^*P <* 0.05, SSPN^−/−^ CD (young vs. aged) mice. Significant groups: Fat (%): ****WT CD vs. HFD (young), **SSPN^−/−^ CD vs. HFD (young), **SSPN^−/−^ HFD (young vs. aged), ****WT CD vs. HFD (aged), ****SSPN^−/−^ CD vs. HFD (aged), ***WT HFD vs. SSPN^−/−^ HFD (young); Lean (%): ****WT CD vs. HFD (young), *SSPN^−/−^ CD vs. HFD (young), ***SSPN^−/−^ HFD (young vs. aged), ****WT CD vs. HFD (aged), ***WT HFD vs. SSPN^−/−^ HFD (young).

Examination of female mouse groups by EchoMRI revealed that young WT female mice had a significant increase in percent fat after HFD that was not seen in SSPN^−/−^ mice (Fig. 3*B*). Aging did influence the response of SSPN^−/−^ female mice to HFD, as they had a significant increase in percent fat, which was not observed in young mice (Fig. 3*B*). Regarding percent lean measurements, HFD female mice of both genotypes had lower percent lean mass than CD controls, which was inversely proportional to their percent fat values (Fig. 3, *C* and *D*). Overall, young SSPN^−/−^ females were largely protected from weight gain; however, age slightly eroded this protection. After HFD, the tissue weights were compared (shown in Fig. 3*E*) and revealed that young female SSPN^−/−^ mice had significantly lower visceral white adipose tissue (VAT) mass values compared with young WT females. The same trend was also seen in VAT from aged female SSPN^−/−^ mice (Fig. 3*F*). This finding corresponds with the EchoMRI data showing lower percent fat for these mice. In contrast, heart and liver weights were not different between WT and SSPN^−/−^ females in either young or aged groups (Fig. 3, *E* and *F*).

### Assessments of Obesogenic Diet and Sarcospan Deletion on Adipocyte Size

After CD and HFD, the visceral WAT from each group of mice was examined for alterations in adipocyte size to assess whether SSPN deletion influenced fat storage. The wet mount images of WAT obtained from young WT HFD male mice revealed adipocytes of varying sizes (Fig. 4*A*, with smaller adipocytes indicative of dying or newly formed adipocytes, whereas the WAT obtained from the young SSPN^−/−^ HFD male mice contained larger, more uniformly sized adipocytes (Fig. 4, *A* and *C*). In addition, the adipocytes were significantly smaller in WAT from young female SSPN^−/−^ HFD mice compared with WT HFD mice (Fig. 4*A*). Adipocyte diameters were significantly increased in WAT obtained from WT females after HFD compared with WT CD mice (Fig. 4*D*).

**Figure 5.**
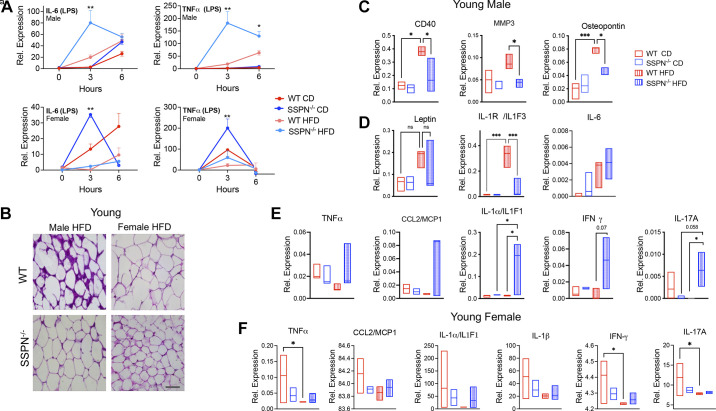
Assessment of white adipose tissue (WAT) inflammation in young sarcospan (SSPN)-deficient (SSPN^−/−^) mice after high-fat diet (HFD). Development of WAT inflammation ultimately involves nonresident macrophage infiltration. *A*: results of in vitro studies assessing the inflammatory response of bone marrow-derived macrophages (BMDMs) to lipopolysaccharide (LPS) (2 mg/mL). BMDMs were obtained from control diet (CD) or HFD young male and female mice. WT, wild type and SSPN^−/−^. *B*: hematoxylin and eosin (H&E)-stained WAT from young male and female WT and SSPN^−/−^ mice after HFD. Images are shown are at ×20 magnification; scale bar, 50 μm. *C*: young male WAT assessed using a proteome profiler cytokine antibody array to detect alterations in adipokines, chemokines, and cytokines; inflammatory markers that were altered in WT male WAT in response to HFD: cluster of differentiation (CD40), matrix metalloproteinase 3 (MMP3), and osteopontin. *D*: leptin, interleukin-1 receptor a/interleukin-1F1 (IL-1Ra/IL1F3), and interleukin-6 (IL-6). *E* and *F*: proinflammatory cytokines plotted that have increased expression in SSPN^−/−^ male WAT (*E*) and results of targeted cytokine panels of WAT obtained from young female mice after exposure to HFD (*F*). Data are shown as floating bars (minimum to maximum), with line indicating the mean. For *A*, data are presented as averages at *time points 0*, *3*, and *6 h*, and each time point was compared and for *C–F*, data were analyzed by 1-way ANOVA followed by Tukey’s post hoc analysis. Different numbers of mice were used: *n* = 2 (*A*), *n* = 4 (*B*), *n* = 3 (*C*), *n* = 3 (*D*), and *n* = 3 or 4 (*E* and *F*). For *E* and *F*, abbreviations used were as follows: CCL2/MCP-1, chemokine (CC-motif) ligand 2/monocyte chemoattractant protein-1; IFN-γ, interferon-γ; IL1α/IL1F1, interleukin-1 alpha/interleukin-1F1; IL-17A, interleukin-17A; IL1F3, interleukin-1F3; TNFα, and tumor necrosis factor-α. Significant values: *P < 0.05, **P < 0.01, and ***P < 0.001; ns, not significant.

In the aged male mouse groups, the WAT was visually similar between WT and SSPN^−/−^ mice with both HFD groups, exhibiting a significant increase in adipocyte diameters compared with controls (Fig. 4, *B* and *E*). The adipocyte diameters from the HFD female aged mice were noticeably smaller than those from HFD male mice (Fig. 5, *B* and *F*). SSPN^−/−^ CD female aged mice had smaller adipocytes compared with WT CD mice. After HFD, however, adipocytes in female aged SSPN^−/−^ mice were larger those of WT HFD mice (Fig. 5*F*).

To follow up previous findings that SSPN variants influenced DNA methylation in the *SSPN* gene promoter region, we assessed the effect of SSPN deletion on global WAT methylation. WAT is normally heavily methylated, and overall we did not see a SSPN-dependent effect on WAT methylation (Supplemental Fig. S3).

### Examining White Adipose Tissue Inflammation in SSPN-Deficient Mice after Diet-Induced Obesity

WAT inflammation involves activation of tissue resident macrophages, and secretion of proinflammatory cytokines attracts additional immune cells including peripheral macrophages to the site of inflammation. One question was whether SSPN deletion and/or HFD conditions influenced the inflammatory response of peripheral macrophages. To address this, bone marrow-derived macrophages (BMDMs) were isolated from young CD or HFD mice. Interestingly, after stimulation with lipopolysaccharide (LPS) that activates Toll-like receptor 4 (TLR4) signaling, a sex-dependent response in proinflammatory cytokine release was detected by qRT-PCR. BMDMs obtained from male SSPN^−/−^ HFD mice exhibited faster kinetics and heightened activation (IL-6 and TNFα release) (Fig. 5*A*, *top*). In contrast, in Fig. 5*A*, *bottom*, BMDMs obtained from female HFD mice exhibited an attenuated response to LPS (lower levels of IL-6 and TNFα release), whereas BMDMs from female SSPN^−/−^ CD mice released significantly higher amounts of TNFα and IL-6 compared with the other groups.

**Figure 6.**
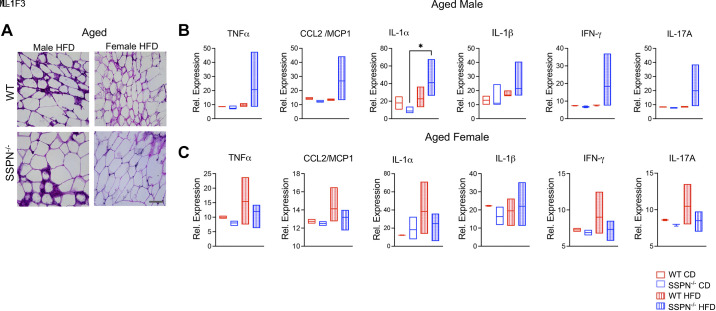
Examination of white adipose tissue (WAT) inflammation in aged mice exposed to high-fat diet (HFD). *A*: hematoxylin & eosin (H&E)-stained WAT sections obtained from aged male and female wild-type (WT) and sarcospan-deficient (SSPN)^−/−^ mice (images at ×20; scale bar, 50 μm). *B* and *C*: abundance of proinflammatory cytokines assessed by cytokine array in WAT obtained from aged males (*B*) and aged females (*C*). Cytokine array data used samples from *n* = 3 or 4 mice. Data are shown as floating bars (minimum to maximum), with line indicating the mean. CCL2/MCP-1, chemokine (CC-motif) ligand 2/monocyte chemoattractant protein-1; CD, control diet; IFN-γ, interferon-γ; IL-1α, interleukin-1α/interleukin-1F1; IL-17A, interleukin-17A; TNFα, tumor necrosis factor-α. Data were analyzed by 1-way ANOVA followed by Tukey’s post hoc analysis. Significance: *P < 0.05.

H&E-stained WAT sections from young male and female HFD mice were examined for signs of inflammation including increased cellularity, an indication of immune cell infiltration (Fig. 5*B*). WAT obtained from the WT male mice showed increased cellularity also seen in SSPN^−/−^ WAT but, however, to a lesser extent. The increased purple staining may indicate fibrosis in the WT male WAT (Fig. 5*B*) and is evidence of a stronger acute response to the obesogenic diet in these mice. The panels in Fig. 5*C* are selected results of adipokine/chemokine/cytokine protein levels in young male WAT using the Proteome Profiler mouse XL cytokine array panel. This group was used to screen for significant changes of cytokine levels in the mice that demonstrated the most pronounced response to HFD. The results are shown for the proinflammatory markers with upregulated expression in WT HFD males including the immune cell marker (CD40), matrix metallopeptidase 3 (MMP3), and osteopontin (Fig. 5*C*), whereas Fig. 5*D* shows the results for leptin, IL-1Ra and IL-6, important modulators of WAT inflammation. IL-1Ra was upregulated in WT HFD WAT (Fig. 5*D*) and has an antagonistic action on IL-1α, which in Fig. 5*E* is upregulated in SSPN^−/−^ male WAT. Several proinflammatory cytokines are upregulated in the SSPN^−/−^ WAT in response to HFD; however, only IL-1α and IL-17A were significantly increased (Fig. 5*E*). In Fig. 5*F*, the WAT cytokine results of young female mice are shown. Overall, SSPN deficiency appeared to have an immunosuppressive effect in young female mice compared with WT. Also, these proinflammatory cytokines were expressed at lower levels in WT HFD compared with CD female mice (Fig. 5*F*). Anti-inflammatory adiponectin was increased in the young female SSPN^−/−^ CD and both HFD groups (data not shown). The panel of cytokines for females was selected based upon the results of the full cytokine panel performed in male mice. Results of the young female groups are shown in Fig. 5*F*, which revealed a slight trend toward elevated multiple proinflammatory cytokines in the SSPN^−/−^ CD WAT.

H&E-stained white adipose tissue from aged mice exhibited some signs of inflammation (Fig. 6*A*). The relative levels of proinflammatory cytokines in the aged male mouse WAT followed similar trends seen in young male mice (Fig. 6*B*). IL-17A expression was no longer significantly elevated, and only IL-1α was significantly higher in WAT from SSPN^−/−^ male mice relative to its CD control. Interestingly, in aged female mice there were no significant changes in the proinflammatory cytokine levels, although most were elevated in WAT from female WT HFD mice (Fig. 6*C*). These results may be due to differential aging effects on the immune system.

**Figure 7.**
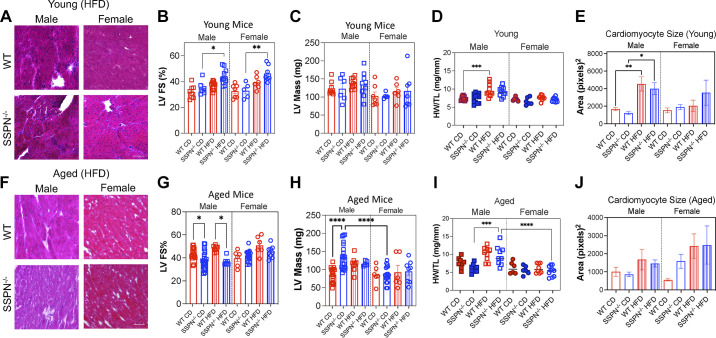
Evaluation of cardiac function of sarcospan (SSPN)-deficient (SSPN^−/−^) mice in response to obesogenic diet. *A* and *F*: transverse wild-type (WT) and SSPN^−/−^ heart sections from young (*A*) and aged (*F*) male and female high-fat diet (HFD) mice were stained with hematoxylin & eosin (H&E). *B* and *C*: echocardiography performed for young control diet (CD) and HFD WT and SSPN^−/−^ mice in left ventricular (LV) fractional shortening (LV FS%; *B*) and LV mass measurements (*C*). *D*: heart weight-to-tibia length ratio (HW/TL) values for young mouse groups. *E*: cardiomyocyte area measurements of young transverse heart sections stained with wheat germ agglutinin (WGA). *G* and *H*: echocardiography for aged mice in LV FS% (*G*) and LV mass (*H*) measurements. *I*: HW/TL measurements are shown for aged mice. *J*: cardiomyocyte areas calculated from WGA-stained hearts. Numbers of mice used for echocardiography measurements in each group are included in Table 5 and Table 6. For cardiac histology and cardiac fiber size (*n* = 3–5) were used. Individual values are shown, and bar height represents the average value. Statistics were calculated by 1-way ANOVA followed by the Tukey’s multiple comparisons post hoc test, *P* < 0.05 was considered significant; errors are reported as means ± SE. Significance: *P < 0.05, **P < 0.01, ***P < 0.001, and ****P < 0.0001.

### Evaluating the Impact of SSPN Deletion and Obesogenic Diet on Cardiac Function of Young and Aged Mice

Echocardiography was used to assess cardiac function in both young and aged WT and SSPN^−/−^ mice after exposure to chronic metabolic stress to assess a role for SSPN in cardiometabolic disease development. We used qRT-PCR to monitor changes in SSPN expression in hearts from female CD and HFD mice (Supplemental Fig. S1). The echocardiography results from young mice are reported in Fig. 7, *B* and *C* and Tables 5 and 6. Overall, cardiac function was preserved in all the groups of mice examined. In Fig. 7*A*, histological evaluation of cardiac tissue from the young HFD mice is shown. However, there was a noticeable increase in cellularity (purple-stained nuclei) after HFD in both male and female SSPN^−/−^ hearts and male WT heart tissue. This may indicate increased immune cell infiltration in the hearts of the HFD mouse groups. H&E-stained aged male heart tissue showed increased interstitial and perivascular fibrosis (Fig. 7*F*). Several SSPN^−/−^ young and aged male hearts exhibited patchy fibrosis; however, this was not seen in all the hearts. Both young male and female SSPN^−/−^ HFD mice had significantly increased left ventricular contractile function with elevated LV ejection fraction (EF)% and LV fractional shortening (FS)% values compared with CD controls (Fig. 7*B* and Tables 5 and 6). In the aged groups, SSPN^−/−^ HFD male mice had slightly reduced LV FS% values compared with WT HFD mice (Fig. 7*G*) and LV EF% values are reported in Table 5.

**Figure 8.**
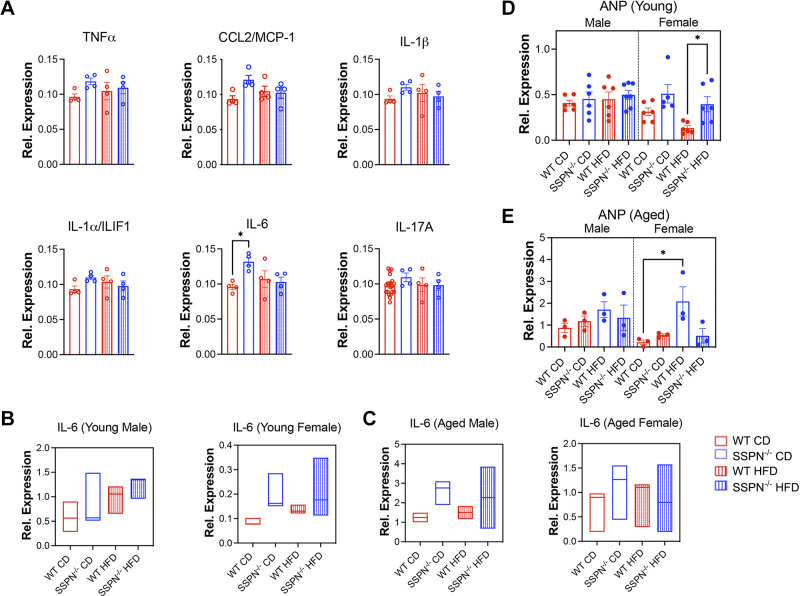
Examination of inflammatory markers in the hearts of young and aged mice in response to obesogenic diet. *A*: cytokine array data of heart tissue are shown from male wild-type (WT) and sarcospan (SSPN)^−/−^ control diet (CD) and high-fat diet (HFD) mice and several cytokines and chemokines reported here. *B* and *C*: quantitative real-time polymerase chain reaction (qRT-PCR) data examine IL-6 levels in heart tissue obtained after CD and HFD regimens for WT and SSPN^−/−^ in young (*B*) and aged (*C*) male and female mice. *D* and *E*: levels of cardiac stress marker atrial natriuretic peptide (ANP) assessed in hearts by qRT-PCR for young (*n* = 5 or 6; *D*) and aged (*n* = 3; *E*) mice. For *A*, *D*, and *E,* individual values are shown, and statistics were calculated by 1-way ANOVA followed by the Tukey’s multiple comparisons post hoc test. For *A*, cytokine array data were from *n* = 4 mice. For *B* and *C*, data are shown as floating bars (minimum to maximum), with line indicating mean (*n* = 3 or 4). For *D* and *E*, *n* = 3. For all groups, *P* < 0.05 was considered significant; errors are reported as means ± SE. Significance: *P < 0.05. CCL2/MCP-1, chemokine (CC-motif) ligand 2/monocyte chemoattractant protein-1; IL-1α/IL1F1, interleukin-1α/interleukin-1F1; IL-1β, interleukin-1β; IL-6, interleukin-6; IL-17A, interleukin-17A; TNFα, tumor necrosis factor-α.

**Table 5.** Summary of male mouse echocardiography values in control and HFD studies

Echo Parameter	Young				Aged			
	WT CD	SSPN^−/−^ CD	WT HFD	SSPN^−/−^ HFD	WT CD	SSPN^−/−^ CD	WT HFD	SSPN^−/−^ HFD
*n*	8	6	10	9	20	24	9	6
HR, beats/min	449.9 ± 15.8	480.2 ± 18.6	460.58 ± 10.4	455.15 ± 20.2	460.6 ± 10.8	449.4 ± 9.9	515.3 ± 4.1	432.4 ± 12.9
LVID;s, mm	2.53 ± 0.13	2.40 ± 0.16	2.45 ± 0.08	1.95 ± 0.12	1.85 ± 0.04^a^	2.12 ± 0.07	1.95 ± 0.05	2.20 ± 0.08
LVID;d, mm	3.69 ± 0.11	3.69 ± 0.15	3.89 ± 0.10	3.47 ± 0.12	3.30 ± 0.07	3.44 ± 0.09	3.73 ± 0.13	3.43 ± 0.09
LV;s, µL	19.00 ± 3.53	20.50 ± 3.32	23.05 ± 1.93	13.27 ± 2.54	10.64 ± 0.62	15.47 ± 1.24	12.06 ± 0.81	16.00 ± 1.70
LV;d, µL	54.61 ± 5.02	61.41 ± 6.10	68.75 ± 5.80	56.13 ± 6.53	44.55 ± 2.10	49.80 ± 2.91	59.97 ± 4.57	48.94 ± 2.97
LV EF, %	60.44 ± 2.58	64.98 ± 2.90	67.58 ± 1.33	75.80 ± 2.28^c^	75.80 ± 1.25^a^	68.58 ± 1.86	80.29 ± 0.7	67.79 ± 1.99^e^
LV FS, %	31.92 ± 1.81	35.14 ± 2.24	37.21 ± 1.04	44.17 ± 2.56^c^	43.76 ± 1.14^a^	38.01 ± 1.54	48.14 ± 0.82	36.96 ± 1.48
SV, µL	34.82 ± 1.63	37.57 ± 2.88	44.37 ± 2.30	37.85 ± 2.68	33.21 ± 1.79	34.28 ± 2.43	47.92 ± 3.93^b^	32.94 ± 1.34^e^
CO, µL	14.09 ± 2.27	17.82 ± 3.57	20.34 ± 1.08	17.15 ± 1.43	15.14 ± 1.10	15.00 ± 1.23	24.67 ± 1.95	14.77 ± 1.02^e^
MV *E/A*	1.58 ± 0.29	1.86 ± 0.66	1.56 ± 0.15	1.65 ± 0.19	1.23 ± 0.05	1.43 ± 0.09	1.34 ± 0. 32	1.33 ± 0.05
IVRT, ms	16.23 ± 1.26	14.94 ± 0.83	14.63 ± 0.98	18.18 ± 1.41	19.88 ± 1.32	16.10 ± 0.96^h^	16.24 ± 1.73^a^	22.76 ± 0.77^g,i^
IVCT, ms	15.00 ± 0.01	17.16 ± 1.17	14.17 ± 0.01	15.84 ± 0.01	13.69 ± 0.96	17.00 ± 1.95	16.92 ± 2.75	16.42 ± 1.1
LV_mass_, mg	121.8 ± 7.20	119.10 ± 16.28	111.25 ± 2.92	107.96 ± 9.52	107.6 ± 5.74	165.6 ± 9.23^d^	144.3 ± 8.27	131.1 ± 6.72
Age, mo	8.42 ± 0.40	6.99 ± 0.24	7.22 ± 0.09	7.64 ± 0.31	18.85 ± 0.68	17.20 ± 0.15	16.43 ± 0.00	17.39 ± 0.10
HW/TL, mg/mm	7.45 ± 0.20	7.42 ± 0.37	9.65 ± 0.56	9.21 ± 0.42	6.89 ± 0.77	6.45 ± 0.39	8.68 ± 0.69	10.58 ± 1.17^g^

Values are means ± SE; *n*, number of mice. CD, control diet; CO, cardiac output; Echo, echocardiography; EF, ejection fraction; FS, fractional shortening; HFD, high-fat diet; HR, heart rate; HW/TL, heart weight-to-tibia length ratio; ID;d, inner dimension, diastole; ID;s, inner dimension, systole; IVCT, intraventricular contraction time; IVRT, isovolumic relaxation time measurements; LV, left ventricular; SV, stroke volume; MV *E/A*, mitral-valve *E/A*; SSPN^−/−^, sarcospan deficient; WT, wild type. One-way ANOVA was used to compare differences between groups. ^a^*P* < 0.05, WT (young vs. aged) CD mice; ^b^*P* < 0.05, WT (young vs. aged) HFD mice; ^c^*P* < 0.05, SSPN^−/−^ (young) CD vs. HFD mice; ^d^*P* < 0.05, WT vs. SSPN^−/−^ (aged) CD mice; ^e^*P* < 0.05, WT vs. SSPN^−/−^ (aged) HFD mice; ^f^*P* < 0.05, WT CD vs. HFD (aged) mice; ^g^*P* < 0.05, SSPN^−/−^ (aged) CD vs. HFD mice; ^h^*P* < 0.05, SSPN^−/−^ (young vs. aged) CD mice; ^i^*P* < 0.05, SSPN^−/−^ (young vs. aged) HFD mice. Significant groups: WT CD male (young vs. aged): ****LVID;s, ****LV EF%, ****LV FS%, **CO; HFD WT vs. SSPN^−/−^ (aged): *CO; CD WT vs. KO (aged): ****LV_mass_; WT CD vs. HFD (aged): **SV, *IVRT; HFD WT (young vs. aged): *LV EF%, *LV FS%; SSPN^−/−^ CD vs. HFD (young): *LV EF%, * LV FS%; SSPN^−/−^ (young vs. aged) CD mice: **IVRT; SSPN^−/−^ (young vs. aged) HFD mice: *IVRT.

**Table 6.** Summary of female mouse echocardiography values in control and HFD studies

Echo Parameter	Young				Aged			
	WT CD	SSPN^−/−^ CD	WT HFD	SSPN^−/−^ HFD	WT CD	SSPN^−/−^ CD	WT HFD	SSPN^−/−^ HFD
*n*	7	5	6	7	7	18	6	8
HR, beats/min	461.7 ± 21.1	429.9 ± 10.4	460.92 ± 11.0	445.43 ± 19.7	531.8 ± 34.4	477.0 ± 12.0	509.5 ± 22.0	482.2 ± 10.2
LVID;s, mm	2.23 ± 0.16	2.40 ± 0.22	1.80 ± 0.08	1.85 ± 0.04	1.83 ± 0.20	1.95 ± 0.06	1.44 ± 0.15	1.83 ± 0.09
LVID;d, mm	3.44 ± 0.17	3.56 ± 0.22	3.47 ± 0.18	3.28 ± 0.07	3.20 ± 0.17	3.33 ± 0.06	2.88 ± 0.10^a^	3.34 ± 0.06
LV;s, µL	11.62 ± 1.15	14.21 ± 1.97	16.27 ± 2.16	8.96 ± 1.98	13.36 ± 3.14	12.92 ± 1.15	6.89 ± 2.39	15.14 ± 1.99
LV;d, µL	44.53 ± 3.51	48.82 ± 1.65	55.06 ± 5.12	40.34 ± 2.65	41.83 ± 4.86	45.68 ± 1.85	32.59 ± 3.17^a^	45.63 ± 1.88
LV EF, %	61.74 ± 2.89	62.09 ± 3.76	70.61 ± 2.56	78.18 ± 2.30^c^	74.98 ± 4.09	72.99 ± 1.35	82.40 ± 3.30	76.6 ± 2.57
LV FS, %	32.6 ± 1.98	32.99 ± 2.60	39.39 ± 2.16	46.31 ± 2.37^c^	39.64 ± 2.80	41.47 ± 1.12	50.88 ± 3.26	45.3 ± 1.96
SV, µL	30.41 ± 3.10	32.97 ± 3.84	36.06 ± 4.71	36.59 ± 3.32	32.29 ± 2.93	32.79 ± 1.01	25.71 ± 0.95	35.1 ± 0.98
CO, µL	12.64 ± 2.17	14.30 ± 1.83	16.61 ± 2.30	14.58 ± 2.07	16.18 ± 2.00	14.77 ± 1.02	12.94 ± 0.71	17.0 ± 0.71
MV *E/A*	1.97 ± 0.26	1.62 ± 0.11	2.28 ± 0.19	2.27 ± 0.13	1.46 ± 0.10	1.31 ± 0.19	1.34 ± 0.14	1.33 ± 0.09^b^
IVRT, ms	16.05 ± 0.75	13.92 ± 1.10	18.31 ± 1.95	18.17 ± 1.88	15.17 ± 1.23	15.70 ± 1.46	14.10 ± 1.04	15.31 ± 0.70
IVCT, ms	13.10 ± 1.20	11.85 ± 1.11	11.19 ± 0.01	11.97 ± 0.01	16.18 ± 2.50	10.41 ± 0.56	13.37 ± 2.18	16.42 ± 1.12
LV_mass_, mg	103.0 ± 13.16	103.5 ± 4.70	108.4 ± 9.51	107.6 ± 5.74	104.6 ± 13.13	109.4 ± 6.60	116.1 ± 22.92	119.1 ± 13.45
Age, mo	8.41 ± 0.40	8.35 ± 0.50	7.74 ± 0.25	8.12 ± 0.51	17.43 ± 0.55	17.20 ± 0.15	18.69 ± 0.40	17.17 ± 0.19
HW/TL, mg/mm	7.19 ± 0.21	6.62 ± 0.42	7.77 ± 0.17	7.08 ± 0.19	6.25 ± 0.68	6.34 ± 0.84	7.06 ± 1.39	6.19 ± 0.41

Values are means ± SE; *n*, number of mice. CD, control diet; CO, cardiac output; Echo, echocardiography; EF, ejection fraction; FS, fractional shortening; HFD, high-fat diet; HR, heart rate; HW/TL, heart weight-to-tibia length ratio; ID;d, inner dimension, diastole; ID;s, inner dimension, systole; IVCT, intraventricular contraction time; IVRT, isovolumic relaxation time measurements; LV, left ventricular; SV, stroke volume; MV *E/A*, mitral valve *E/A*; SSPN^−/−^, sarcospan deficient; WT, wild type. One-way ANOVA was used to compare differences between groups. ^a^*P* < 0.05, WT (young vs. aged) HFD mice; ^b^*P* < 0.05, SSPN^−/−^ (young vs. aged) HFD mice; ^c^*P* < 0.05, SSPN^−/−^ (young) CD vs. HFD mice. Significant groups: WT (young vs. aged) HFD mice: *LVID;s, *LV;d; SSPN^−/−^ (young vs. aged) HFD mice: *E/A, IVRT; SSPN^−/−^ (young) CD vs. HFD mice: *LV EF%, *LV FS%), **; WT vs. SSPN^−/−^ aged CD mice, WT (aged) CD vs. HFD mice.

The mouse groups were evaluated for changes in heart size and ventricular mass to assess cardiac remodeling in response to metabolic stress and weight gain. Heart weight-to-tibia length ratio (HW/TL) measurements are shown for young mice in Fig. 7*D* and for aged mice in Fig. 7*I*. In young mouse groups, only the male WT HFD mice exhibited a significant increase in HW/TL. Comparisons were made between LV mass (Fig. 7*C*), overall heart size (HW/TL) (Fig. 7*D*), and cardiomyocyte area in wheat germ agglutinin (WGA)-stained hearts to determine whether changes in heart size could be attributed to cardiomyocyte hypertrophy. Young male WT mice had increased HW/TL values after HFD corresponding to increased cardiomyocyte hypertrophy (Fig. 7*E*). In the aged mouse groups, the CD SSPN^−/−^ mice had a significant increase in LV mass (Fig. 7*H*), which suggests concentric remodeling since the HW/TL was not increased and no evidence of cardiomyocyte hypertrophy was found (Fig. 7*J*). Aged HFD SSPN^−/−^ mice had a significant increase in HW/TL compared with their respective CD control, with, however, no change in LV mass or cardiomyocyte area (Fig. 7, *H*–*J*), whereas aged female SSPN^−/−^ mice did not show signs of cardiac hypertrophy with age or metabolic stress after HFD (Fig. 7, *H*–*J*).

Since metabolic-derived cardiac dysfunction typically first manifests as diastolic dysfunction, the mice were also assessed by mitral valve Doppler echocardiography (Table 5 and Table 6). The ratio between early and late ventricular filling is represented by *E/A*, which was elevated in the young female HFD groups of mice; *E/A* values higher than 1.0 may reflect a degree of diastolic function in mice. However, other factors can influence *E/A* values, including alterations in left atrial pressure. Therefore, isovolumic relaxation time (IVRT) was also reported to complement the *E/A* findings. In the young female SSPN^−/−^ HFD mice, IVRT was also increased (Table 6). These findings suggest that the young female mice showed signs of developing diastolic dysfunction at a young age; however, at later ages it had not progressed. This could indicate that both the *E/A* and IVRT were being influenced by other factors. Since both *E/A* and IVRT can be influenced by atrial pressures, these conclusions should be reevaluated using tissue Doppler approaches. On the other hand, young SSPN^−/−^ male HFD mice had increased *E/A* and IVRT values (Table 5). The IVRT values were further prolonged in aged SSPN^−/−^ mice showing progression of diastolic dysfunction.

### Assessing Inflammation and Stress Markers in Hearts of SSPN-Deficient Mice after Diet-Induced Obesity

**Figure 9.**
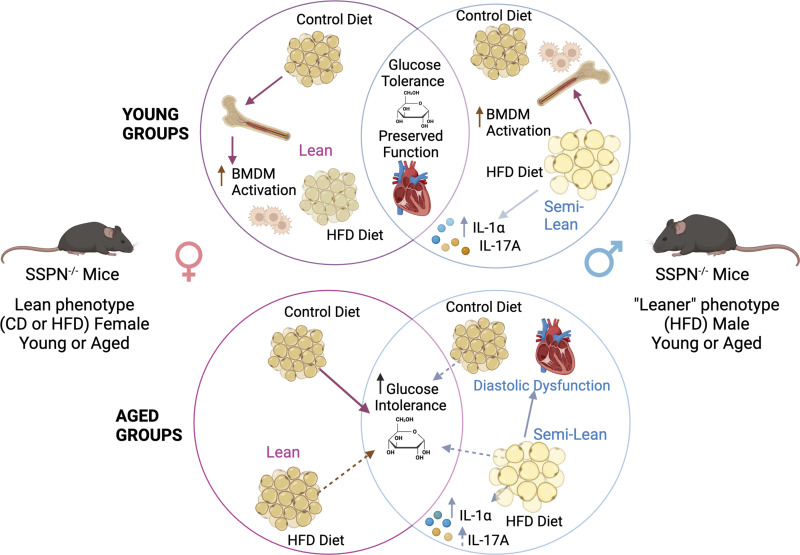
Summary of major findings in sarcospan (SSPN)-deficient (SSPN^−/−^) mice exposed to chronic metabolic stress. *Top* circles show results from the young SSPN^−/−^ mice, pink circle representing findings in females and the intersecting area showing common phenotypes between females and males. Impact of sex and diet is depicted by altered adipocyte size. *Bottom* circles show results from aged groups of SSPN^−/−^ mice. For purposes of simplification, the results for wild-type (WT) mice are not shown. Solid arrows indicate significant findings, whereas dashed arrows indicate trends. BMDM, bone marrow-derived macrophage; CD, control diet; HFD, high-fat diet; IL-1α, interleukin-1α; IL-17A, interleukin-17A. Created with a licensed version of BioRender.com.

Cardiac tissue was assessed for signs of inflammation after HFD diet and upon aging. The results of select proinflammatory cytokines are shown in (Fig. 8*A*), and no differences were noted in the young male HFD mouse groups. In the CD groups, only IL-6 was significantly increased in SSPN^−/−^ male hearts. qRT-PCR was used to assess IL-6 cytokine levels in young mouse hearts (Fig. 8*B*), and results are shown from aged hearts in Fig. 8*C*. Atrial natriuretic peptide (ANP) levels are used as a cardiac stress marker and were found increased in young female HFD SSPN^−/−^ hearts compared with HFD WT (Fig. 8*D*), and in aged mice they were increased in female WT HFD mice compared with their control (Fig. 8*E*). A summary of the major findings in SSPN^−/−^ mice during this study is shown in Fig. 9.

## DISCUSSION

This study follows up on previous reports that SSPN plays a role in fat distribution in the body (46). The implication is that SSPN influences metabolic pathways that result in altered fat distribution (46). In this study, we examined whether the absence of SSPN impacts white adipose tissue homeostasis and cardiac function over the lifetime of SSPN-deficient mice. Sex differences have also been reported in human studies, so we also examined them in our study. Overall, loss of the SSPN protein partially protected both young male and female mice from weight gain, with a stronger effect in female mice even after aging. Extrapolating aging results from mice to humans can be difficult in terms of differences in lifespan but also alterations in sex hormones. Limitations exist when using female mice to recapitulate aging effects in humans because of differences in estrogen effects, where at equivalent ages women are in menopause as estrogen levels decline after the age of 50 while mice undergo estropause manifesting with enhanced estrogen levels (47). After >13 mo of age, C57BL/6J female mice have decreased cycling; however, estrogen levels remain similar to young C57BL/6J mice (48, 49). Therefore, additional measures such as ovariectomy are required to reduce estrogen levels so that aged female mice more closely approximate human female menopause.

Additional factors should be considered when equating SSPN deficiency with lower body weights in mice. The control mice were fed a standard chow fed (4%), which is lower than commonly used standard chow diets. Diet and consumption differences may contribute to lower weight gain seen in SSPN^−/−^ HFD mice in our study. Interestingly, in this study all SSPN^−/−^ CD groups except for young males had an increased percent change in body weight compared with WT mice. In an earlier study, male and female SSPN^−/−^ mice on a mixed (C57BL/6–129 SV/J) background were fed ad libitum with a higher-energy concentration NIH-31-modified rodent diet (5% fat) and found to be heavier than their WT counterparts (50). The changes in HFD food consumption in aged SSPN^−/−^ mice may be influenced by heightened changes in glutamatergic nerve cell activity that occur in the lateral hypothalamus of mice fed HFD over a longer time period later in life (51, 52).

At a young age the SSPN^−/−^ mice had normal abilities to clear glucose in response to glucose tolerance testing even after 4 mo of obesogenic diet. Aging, inflammation, and metabolic stress all undermine the normal insulin sensitivity of tissues, impairing glucose uptake. It has been documented that C57BL/6J mice remain more insulin sensitive than other strains despite higher weight gain in response to diet-induced obesity (53). Another study showed that male C57BL/6J mice experience a significant deterioration in glucose tolerance from 6 to 18 mo of age not observed in female mice (54). In contrast, our study used mice from 6 to 18 mo of age, and we did not observe significant alterations in the AUC after glucose administration in aged male CD WT mice. This difference may be due to the glucose dose (1 g/kg) in our study compared with 2 g/kg in the previously referenced study.

An unexpected finding was the effect of aging on the glucose tolerance of SSPN^−/−^ compared with WT CD mice. On a low-fat standard chow diet, the aged SSPN mice developed glucose intolerance compared with WT mice (Fig. 2, *B* and *D*; Table 1 and Table 2). Aged CD and HFD SSPN^−/−^ females were lean but had high fasting blood glucose values in the diabetic range and had significantly poorer glucose clearance compared with CD females. This suggests that the SSPN^−/−^ mice were metabolically unhealthy compared with WT mice with a poor tissue response to insulin. They had a higher percent change in body weight compared with WT controls (Fig. 1, *G* and *H*); however, overall, both males and females were leaner and had lower overall percent body fat (Fig. 3, *A* and *B*) and smaller visceral WAT fat pads (Fig. 3, *E* and *F*). The aged SSPN^−/−^ female mice may have had a gradual change in body fat distribution, shifting from the more protective subcutaneous to visceral fat pad storage, which can contribute to increased metabolic risk including insulin resistance (55–57).

One obvious explanation for the aging-related glucose intolerance of SSPN^−/−^ female mice is that estrogen levels may have been differentially affected in the SSPN^−/−^ female mice, rendering them more susceptible to metabolic dysfunction. Since blood estrogen concentrations were not measured, a decline in estrogen cannot be ruled out in the aged SSPN^−/−^ mice. Other possible explanations include a reduction in pancreatic β-cell function seen in lean individuals who develop “lean” diabetes (58). The glucose intolerance observed in lean CD SSPN^−/−^ mice may rely on unique pathophysiological mechanisms related to SSPN deficiency and development of lean diabetes (58). The smaller size of SSPN^−/−^ mice has been noted before 2 mo of age, especially in females. It is not known if SSPN^−/−^ males and females have birth weights different from corresponding WT. The smaller size could also signal underlying metabolic dysfunction, and low birth weight has been associated with increased risk of metabolic syndrome and increased susceptibility to insulin resistance (59–61). In human association studies, SNPs in the *SSPN* locus had strong associations with increased waist-to-hip ratio (WHR) (62), fasting insulin adjusted for body mass index (FIadjBMI), and type 2 diabetes (10). These SNPs appear to alter essential functions of SSPN or affect its expression (10).

Another source of insulin resistance results from the inflammation that develops in WAT during the development of obesity. Visual examination of WAT from young male mice revealed obvious signs of inflammatory activity and fibrosis (Fig. 5*B*). To further address this, cytokine panels were run to assess the degree of WAT inflammation in the different mouse groups in the study. Changes in the adipose microenvironment can be detected by examining alterations in proinflammatory or anti-inflammatory cytokines or molecules. This can provide an overview of underlying immune cell populations in the tissue and potential for immune cell recruitment. In young males after HFD, the levels of typically assessed cytokines were generally higher in WT compared with SSPN^−/−^. In addition to the fibrosis, it appeared that active extracellular matrix (ECM) remodeling was active in the young WT male WAT after HFD since MMP3 and osteopontin (OPN) were elevated (63, 64). Increased CD40 levels are indicative of adaptive immune cell involvement by CD40-expressing cells (65). Most OPN in adipose tissue (AT) is expressed by AT macrophages, which are important in regulating local inflammation and cell death. In mouse obesity studies, OPN has been found to be the principal cytokine overexpressed in adipose tissue (66).

In contrast to results in WT HFD male mice, SSPN deficiency appeared to contribute to chronic higher levels of WAT inflammation in response to diet-induced obesity. In SSPN^−/−^ WAT, higher levels of proinflammatory cytokine were detected, with significant elevations in IL-1α and IL-17A. Bone marrow-derived inflammatory cells are key drivers of low-grade inflammation, termed WAT metainflammation (67, 68). The best-characterized population of immune cells involved in the response to obesity are adipose tissue (AT) macrophages. AT macrophages are the main source of proinflammatory mediators such as TNFα, MCP-1 and IL-6 (69, 70) that have been documented to contribute to metabolic dysfunction in obese individuals (67, 71, 72) and development of insulin resistance in type 2 diabetes (63). Infiltrating macrophages can also shape the WAT environment in obesity. Our results from LPS-stimulated BMDMs obtained from young mice illustrate this and may underscore the distinct sex differences seen in response to HFD. The BMDMs from female HFD mice had an attenuated response to LPS (Fig. 5*A*) and had lower levels of AT inflammation in response to HFD (Fig. 5*F*). The predisposition of SSPN^−/−^ female mice toward insulin resistance may further dysregulate inflammatory signaling of AT macrophages that examined the response of BMDMs obtained from diabetic mice (73).

The pathology of type 2 diabetes is influenced by IL-1 activity, which mediates obesity-induced inflammation with a direct influence on insulin resistance (74). VAT from obese compared with lean individuals has higher levels of IL-1Ra. IL-1α acts as a dual-function cytokine and can translocate to the nucleus as a transcription factor for proinflammatory genes including IL-1 and IL-6 (74). The role of the proinflammatory cytokine IL-1α is less clear in obesity; however, it is thought to recruit innate immune cells to AT in response to necrotic adipocyte release of “danger signals.” IL-1α may control early steps of AT inflammation during the onset of obesity and is antagonized by endogenous levels of IL-1 receptor antagonist (IL-1Ra) to specifically inhibit IL-1 signaling (75). Sustained activation of IL-1α, however, stimulates adaptive immune functions and can diminish Treg suppressive function (76). In our study the significant increase of IL-1α in SSPN^−/−^ after chronic exposure to obesity-promoting conditions and heightened levels of its regulator IL-1Ra in WT may underlie the temporal differences in WAT inflammation seen between these groups.

Also of note was the observation that the proinflammatory cytokine IL-17A was upregulated in the WAT of young and aged SSPN^−/−^ male mice after HFD (Figs. 5*E* and Fig. 6*B*). The family of IL-17 cytokines have protective roles against infectious agents, promote recruitment of neutrophils, and, when dysregulated, can contribute to many autoimmune and chronic inflammatory disorders (77). The primary source of IL-17 in adipose tissue is γδ T cells, but it can be produced by other cell types including neutrophils. As obesity progresses, neutrophils are the earliest cells to infiltrate into WAT. IL-17 has also been found to act as a negative regulator of adipogenesis and glucose metabolism in mice and overall delay the development of obesity, and IL-17-deficient mice have increased susceptibility to diet-induced obesity (78). Future studies involving antibody-based neutralization are required to determine whether protection from obesity is diminished when these pathways are blocked.

Examination of cardiac function in SSPN^−/−^ mice is important to determine whether SSPN contributes to cardiometabolic disease development. Recapitulating obesity-driven cardiac dysfunction in mouse models is challenging because mice maintain relative normal heart function with age and long-duration HFD studies (79). Echocardiography was used to examine the mice in the present study, and overall no decrements were observed in systolic parameters in any of the mouse groups. One limitation of our study is that data are not presented in a way that illustrates cardiac function changes that occur in each mouse in response to the diet intervention. Cardiac remodeling was primarily observed in male groups after HFD and appeared to be the result of hypertrophic heart growth resulting from cardiomyocyte hypertrophy (Fig. 7*E*). Cardiac hypertrophy in response to HFD has been well documented in C57BL/6J mice (80) and was observed in the young male WT HFD mice, which also had the greatest increase in body mass. Aging led to a significant increase in LV_mass_ in aged SSPN^−/−^ male mice under baseline CD conditions and was likely a result of concentric hypertrophy since no cardiac dysfunction or chamber dilation was detected. This supports earlier studies that indicate that the SSPN locus plays a distinct role in determining cardiac mass (32). It does suggest that the hearts of these mice have undergone compensatory adaptations to physiological stressors over their lifetime and should be investigated further.

Mild diastolic dysfunction occurs in response to obesity and becomes more severe as metabolic function becomes more deranged. Females are more prone to diastolic dysfunction related to metabolic disorders (81). Impaired cardiac relaxation can be detected as a prolongation of IVRT that increases in diabetic cardiomyopathy and aging (82). In healthy WT mice, normal IVRT values were reported around 17.5 mo of age (83). Addition of metabolic stress caused the aged male SSPN^−/−^ mice to develop diastolic dysfunction by the end of the HFD study. The impaired relaxation was evident in their prolonged IVRT values (Table 5). The *E/A* ratios of both aged female and male mice were largely within the normal range (Table 5 and Table 6) but should be interpreted cautiously as a measure of diastolic dysfunction. As the SSPN^−/−^ male mice exhibited increased WAT inflammation, it is possible they have higher levels of circulating proinflammatory molecules that damage the heart and contribute to the development of cardiac dysfunction (84). However, there were minimal changes in the inflammatory environment of the heart, although IL-6 levels were significantly increased in young male CD SSPN^−/−^ hearts (Fig. 8*A*). Future studies are needed to understand factors that may contribute to cardiac remodeling in aging and metabolically challenged SSPN^−/−^ mice. In this study mitral flow-Doppler echocardiography was solely used to assess changes in diastolic function. Tissue Doppler will be used in future studies to follow the progression of diastolic dysfunction over a time course since it is more sensitive and less dependent on external factors.

In conclusion, SSPN deficiency in mice results in a lean phenotype under obesogenic conditions with accompanying glucose intolerance in both sexes of mice regardless of diet. In the high-fat diet study, sex-specific differences were observed in male mice that had chronic white adipose tissue inflammation and with aging developed diastolic dysfunction.

## DATA AVAILABILITY

Data will be made available upon reasonable request.

## SUPPLEMENTAL MATERIAL

10.6084/m9.figshare.26048563.v2 Supplemental Figs. S1–S3: https://doi.org/10.6084/m9.figshare.26048563.v2.

## GRANTS

This study was funded in part by American Heart Association Scientist Development Grant 16SDG29120002 and Florida Department of Health’s James and Esther King Biomedical Research Program Grant 21K12.

## DISCLAIMERS

The content here within is solely the authors’ responsibility and does not necessarily represent the official views of the Florida State University.

## DISCLOSURES

No conflicts of interest, financial or otherwise, are declared by the authors.

## AUTHOR CONTRIBUTIONS

I.C.V. and M.S.P. conceived and designed research; A.R., I.C.V., R.Q.C., L.S., G.R., S.E., N.M., B.S.O., A.R.M., and M.S.P. performed experiments; A.R., I.C.V., R.Q.C., L.S., G.R., R.M.K., and M.S.P. analyzed data; A.R. and M.S.P. interpreted results of experiments; A.R., L.S. and, M.S.P. prepared figures; M.S.P. drafted manuscript; A.R., I.C.V., R.M.K., and M.S.P. edited and revised manuscript; A.R., I.C.V., R.Q.C., L.S., S.E., R.M.K., N.M., B.S.O., A.R.M., and M.S.P. approved final version of manuscript.

## References

[1] Finucane MM, Stevens GA, Cowan MJ, Danaei G, Lin JK, Paciorek CJ, Singh GM, Gutierrez HR, Lu Y, Bahalim AN, Farzadfar F, Riley LM, Ezzati M; Global Burden of Metabolic Risk Factors of Chronic Diseases Collaborating Group. National, regional, and global trends in body-mass index since 1980: systematic analysis of health examination surveys and epidemiological studies with 960 country-years and 9.1 million participants. Lancet 377: 557–567, 2011. doi:10.1016/S0140-6736(10)62037-5. 21295846 PMC4472365

[2] Ferrannini E, Iozzo P. Is insulin resistance atherogenic? A review of the evidence. Atheroscler Suppl 7: 5–10, 2006. doi:10.1016/j.atherosclerosissup.2006.05.006. 16824807

[3] Fox A, Feng W, Asal V. What is driving global obesity trends? Globalization or “modernization”? Global Health 15: 32, 2019. doi:10.1186/s12992-019-0457-y. 31029156 PMC6486955

[4] Srivastava AK. Challenges in the treatment of cardiometabolic syndrome. Indian J Pharmacol 44: 155–156, 2012. doi:10.4103/0253-7613.93579. 22529466 PMC3326903

[5] Yang W, Kelly T, He J. Genetic epidemiology of obesity. Epidemiol Rev 29: 49–61, 2007. doi:10.1093/epirev/mxm004. 17566051

[6] Liu CT, Buchkovich ML, Winkler TW, Heid IM, African Ancestry Anthropometry Genetics Consortium, GIANT Consortium, Borecki IB, Fox CS, Mohlke KL, North KE, Cupples LA. Multi-ethnic fine-mapping of 14 central adiposity loci. Hum Mol Genet 23: 4738–4744, 2014. doi:10.1093/hmg/ddu183. 24760767 PMC4119415

[7] Heid IM, Jackson AU, Randall JC, Winkler TW, Qi L, Steinthorsdottir V, , et al Meta-analysis identifies 13 new loci associated with waist-hip ratio and reveals sexual dimorphism in the genetic basis of fat distribution. Nat Genet 42: 949–960, 2010 [Erratum in *Nat Genet* 43: 1164, 2011]. doi:10.1038/ng.685. 20935629 PMC3000924

[8] Fox CS, Liu Y, White CC, Feitosa M, Smith AV, Heard-Costa N, Lohman K, GIANT Consortium, MAGIC Consortium, GLGC Consortium, Johnson AD, Foster MC, Greenawalt DM, Griffin P, Ding J, Newman AB, Tylavsky F, Miljkovic I, Kritchevsky SB, Launer L, Garcia M, Eiriksdottir G, Carr JJ, Gudnason V, Harris TB, Cupples LA, Borecki IB. Genome-wide association for abdominal subcutaneous and visceral adipose reveals a novel locus for visceral fat in women. PLoS Genet 8: e1002695, 2012. doi:10.1371/journal.pgen.1002695. 22589738 PMC3349734

[9] Winkler TW, Justice AE, Graff M, Barata L, Feitosa MF, Chu S, , et al The influence of age and sex on genetic associations with adult body size and shape: a large-scale genome-wide interaction study. PLoS Genet 11: e1005378, 2015 [Erratum in *PLoS Genet* 12: e1006166, 2016]. doi:10.1371/journal.pgen.1005378. 26426971 PMC4591371

[10] Shungin D, Winkler TW, Croteau-Chonka DC, Ferreira T, Locke AE, Magi R, , et al New genetic loci link adipose and insulin biology to body fat distribution. Nature 518: 187–196, 2015. doi:10.1038/nature14132. 25673412 PMC4338562

[11] Turcotte M, Abadi A, Peralta-Romero J, Suarez F, Reddon H, Gomez-Zamudio J, Burguete-Garcia AI, Cruz M, Meyre D. Genetic contribution to waist-to-hip ratio in Mexican children and adolescents based on 12 loci validated in European adults. Int J Obes (Lond) 43: 13–22, 2019. doi:10.1038/s41366-018-0055-8. 29777226

[12] Graff M, Fernández-Rhodes L, Liu S, Carlson C, Wassertheil-Smoller S, Neuhouser M, Reiner A, Kooperberg C, Rampersaud E, Manson JE, Kuller LH, Howard BV, Ochs-Balcom HM, Johnson KC, Vitolins MZ, Sucheston L, Monda K, North KE. Generalization of adiposity genetic loci to US Hispanic women. Nutr Diabetes 3: e85, 2013. doi:10.1038/nutd.2013.26. 23978819 PMC3759132

[13] Peter AK, Marshall JL, Crosbie RH. Sarcospan reduces dystrophic pathology: stabilization of the utrophin-glycoprotein complex. J Cell Biol 183: 419–427, 2008. doi:10.1083/jcb.200808027. 18981229 PMC2575773

[14] Gibbs EM, Marshall JL, Ma E, Nguyen TM, Hong G, Lam JS, Spencer MJ, Crosbie-Watson RH. High levels of sarcospan are well tolerated and act as a sarcolemmal stabilizer to address skeletal muscle and pulmonary dysfunction in DMD. Hum Mol Genet 25: 5395–5406, 2016. doi:10.1093/hmg/ddw356. 27798107 PMC5418831

[15] Marshall JL, Oh J, Chou E, Lee JA, Holmberg J, Burkin DJ, Crosbie-Watson RH. Sarcospan integration into laminin-binding adhesion complexes that ameliorate muscular dystrophy requires utrophin and alpha7 integrin. Hum Mol Genet 24: 2011–2022, 2015. doi:10.1093/hmg/ddu615. 25504048 PMC4355028

[16] Parvatiyar MS, Brownstein AJ, Kanashiro-Takeuchi RM, Collado JR, Dieseldorff Jones KM, Gopal J, Hammond KG, Marshall JL, Ferrel A, Beedle AM, Chamberlain JS, Renato Pinto J, Crosbie RH. Stabilization of the cardiac sarcolemma by sarcospan rescues DMD-associated cardiomyopathy. JCI Insight 5: e123855, 2019. doi:10.1172/jci.insight.123855. 31039133 PMC6629091

[17] Parvatiyar MS, Marshall JL, Nguyen RT, Jordan MC, Richardson VA, Roos KP, Crosbie-Watson RH. Sarcospan regulates cardiac isoproterenol response and prevents Duchenne muscular dystrophy-associated cardiomyopathy. J Am Heart Assoc 4: e002481, 2015. doi:10.1161/JAHA.115.002481. 26702077 PMC4845268

[18] Crosbie RH, Heighway J, Venzke DP, Lee JC, Campbell KP. Sarcospan, the 25-kDa transmembrane component of the dystrophin-glycoprotein complex. J Biol Chem 272: 31221–31224, 1997. doi:10.1074/jbc.272.50.31221. 9395445

[19] Lebakken CS, Venzke DP, Hrstka RF, Consolino CM, Faulkner JA, Williamson RA, Campbell KP. Sarcospan-deficient mice maintain normal muscle function. Mol Cell Biol 20: 1669–1677, 2000. doi:10.1128/MCB.20.5.1669-1677.2000. 10669744 PMC85350

[20] Coral-Vazquez R, Cohn RD, Moore SA, Hill JA, Weiss RM, Davisson RL, Straub V, Barresi R, Bansal D, Hrstka RF, Williamson R, Campbell KP. Disruption of the sarcoglycan-sarcospan complex in vascular smooth muscle: a novel mechanism for cardiomyopathy and muscular dystrophy. Cell 98: 465–474, 1999. doi:10.1016/s0092-8674(00)81975-3. 10481911

[21] Keller M, Klös M, Rohde K, Kruger J, Kurze T, Dietrich A, Schön MR, Gärtner D, Lohmann T, Dressler M, Stumvoll M, Bluher M, Kovacs P, Böttcher Y. DNA methylation of SSPN is linked to adipose tissue distribution and glucose metabolism. FASEB J 22: fj201800528R, 2018. doi:10.1096/fj.201800528R. 29932866

[22] Marshall JL, Chou E, Oh J, Kwok A, Burkin DJ, Crosbie-Watson RH. Dystrophin and utrophin expression require sarcospan: loss of alpha7 integrin exacerbates a newly discovered muscle phenotype in sarcospan-null mice. Hum Mol Genet 21: 4378–4393, 2012. doi:10.1093/hmg/dds271. 22798625 PMC3459462

[23] Lapidos KA, Kakkar R, McNally EM. The dystrophin glycoprotein complex: signaling strength and integrity for the sarcolemma. Circ Res 94: 1023–1031, 2004. doi:10.1161/01.RES.0000126574.61061.25. 15117830

[24] Valera IC, Wacker AL, Hwang HS, Holmes C, Laitano O, Landstrom AP, Parvatiyar MS. Essential roles of the dystrophin-glycoprotein complex in different cardiac pathologies. Adv Med Sci 66: 52–71, 2021. doi:10.1016/j.advms.2020.12.004. 33387942

[25] Rodriguez-Cruz M, Sanchez R, Escobar RE, Cruz-Guzman Odel R, Lopez-Alarcon M, Bernabe Garcia M, Coral-Vazquez R, Matute G, Velazquez Wong AC. Evidence of insulin resistance and other metabolic alterations in boys with Duchenne or Becker muscular dystrophy. Int J Endocrinol 2015: 867273, 2015. doi:10.1155/2015/867273. 26089900 PMC4452344

[26] Strakova J, Kamdar F, Kulhanek D, Razzoli M, Garry DJ, Ervasti JM, Bartolomucci A, Townsend D. Integrative effects of dystrophin loss on metabolic function of the mdx mouse. Sci Rep 8: 13624, 2018. doi:10.1038/s41598-018-31753-3. 30206270 PMC6134145

[27] Groh S, Zong H, Goddeeris MM, Lebakken CS, Venzke D, Pessin JE, Campbell KP. Sarcoglycan complex: implications for metabolic defects in muscular dystrophies. J Biol Chem 284: 19178–19182, 2009. doi:10.1074/jbc.C109.010728. 19494113 PMC2740540

[28] Wren TA, Bluml S, Tseng-Ong L, Gilsanz V. Three-point technique of fat quantification of muscle tissue as a marker of disease progression in Duchenne muscular dystrophy: preliminary study. AJR Am J Roentgenol 190: W8–W12, 2008. doi:10.2214/AJR.07.2732. 18094282

[29] Lodi R, Muntoni F, Taylor J, Kumar S, Sewry CA, Blamire A, Styles P, Taylor DJ. Correlative MR imaging and ^31^P-MR spectroscopy study in sarcoglycan deficient limb girdle muscular dystrophy. Neuromuscul Disord 7: 505–511, 1997. doi:10.1016/s0960-8966(97)00108-9. 9447608

[30] Yamanouchi K, Yada E, Ishiguro N, Hosoyama T, Nishihara M. Increased adipogenicity of cells from regenerating skeletal muscle. Exp Cell Res 312: 2701–2711, 2006. doi:10.1016/j.yexcr.2006.04.014. 16750191

[31] Zhang X, Cupples LA, Liu CT. A fine-mapping study of central obesity loci incorporating functional annotation and imputation. Eur J Hum Genet 26: 1369–1377, 2018. doi:10.1038/s41431-018-0168-5. 29967334 PMC6117358

[32] Della-Morte D, Beecham A, Rundek T, Wang L, McClendon MS, Slifer S, Blanton SH, Di Tullio MR, Sacco RL. A follow-up study for left ventricular mass on chromosome 12p11 identifies potential candidate genes. BMC Med Genet 12: 100, 2011. doi:10.1186/1471-2350-12-100. 21791083 PMC3199748

[33] Steinberger J, Daniels SR, Eckel RH, Hayman L, Lustig RH, McCrindle B, Mietus-Snyder ML; American Heart Association Atherosclerosis, Hypertension, Obesity in the Young Committee of the Council on Cardiovascular Disease in the Young, Council on Cardiovascular Nursing, Council on Nutrition, Physical Activity, and Metabolism. Progress and challenges in metabolic syndrome in children and adolescents: a scientific statement from the American Heart Association Atherosclerosis, Hypertension, and Obesity in the Young Committee of the Council on Cardiovascular Disease in the Young; Council on Cardiovascular Nursing; and Council on Nutrition, Physical Activity, and Metabolism. Circulation 119: 628–647, 2009. doi:10.1161/CIRCULATIONAHA.108.191394. 19139390

[34] Warren HR, Evangelou E, Cabrera CP, Gao H, Ren M, Mifsud B, , et al Genome-wide association analysis identifies novel blood pressure loci and offers biological insights into cardiovascular risk. Nat Genet 49: 403–415, 2017. doi:10.1038/ng.3768. 28135244 PMC5972004

[35] Sandholt CH, Hansen T, Pedersen O. Beyond the fourth wave of genome-wide obesity association studies. Nutr Diabetes 2: e37, 2012. doi:10.1038/nutd.2012.9. 23168490 PMC3408643

[36] Roselli C, Chaffin MD, Weng LC, Aeschbacher S, Ahlberg G, Albert CM, , et al Multi-ethnic genome-wide association study for atrial fibrillation. Nat Genet 50: 1225–1233, 2018. doi:10.1038/s41588-018-0133-9. 29892015 PMC6136836

[37] Dahlman I, Sinha I, Gao H, Brodin D, Thorell A, Rydén M, Andersson DP, Henriksson J, Perfilyev A, Ling C, Dahlman-Wright K, Arner P. The fat cell epigenetic signature in post-obese women is characterized by global hypomethylation and differential DNA methylation of adipogenesis genes. Int J Obes (Lond) 39: 910–919, 2015. doi:10.1038/ijo.2015.31. 25783037

[38] Wang L, Beecham A, Di Tullio MR, Slifer S, Blanton SH, Rundek T, Sacco RL. Novel quantitative trait locus is mapped to chromosome 12p11 for left ventricular mass in Dominican families: the Family Study of Stroke Risk and Carotid Atherosclerosis. BMC Med Genet 10: 74, 2009. doi:10.1186/1471-2350-10-74. 19627612 PMC2724377

[39] Voisin S, Almén MS, Zheleznyakova GY, Lundberg L, Zarei S, Castillo S, Eriksson FE, Nilsson EK, Bluher M, Bottcher Y, Kovacs P, Klovins J, Rask-Andersen M, Schioth HB. Many obesity-associated SNPs strongly associate with DNA methylation changes at proximal promoters and enhancers. Genome Med 7: 103, 2015. doi:10.1186/s13073-015-0225-4. 26449484 PMC4599317

[40] Rohde K, Keller M, la Cour Poulsen L, Blüher M, Kovacs P, Böttcher Y. Genetics and epigenetics in obesity. Metabolism 92: 37–50, 2019. doi:10.1016/j.metabol.2018.10.007. 30399374

[41] Karastergiou K, Smith SR, Greenberg AS, Fried SK. Sex differences in human adipose tissues - the biology of pear shape. Biol Sex Differ 3: 13, 2012. doi:10.1186/2042-6410-3-13. 22651247 PMC3411490

[42] Lindsey ML, LeBlanc AJ, Ripplinger CM, Carter JR, Kirk JA, Hansell Keehan K, Brunt KR, Kleinbongard P, Kassiri Z. Reinforcing rigor and reproducibility expectations for use of sex and gender in cardiovascular research. Am J Physiol Heart Circ Physiol 321: H819–H824, 2021. doi:10.1152/ajpheart.00418.2021. 34524922

[43] Hatzistergos KE, Paulino EC, Dulce RA, Takeuchi LM, Bellio MA, Kulandavelu S, Cao Y, Balkan W, Kanashiro-Takeuchi RM, Hare JM. *S*-nitrosoglutathione reductase deficiency enhances the proliferative expansion of adult heart progenitors and myocytes post myocardial infarction. J Am Heart Assoc 4, 2015. doi:10.1161/JAHA.115.001974. PMC4608081 26178404

[44] Martins AS, Parvatiyar MS, Feng HZ, Bos JM, Gonzalez-Martinez D, Vukmirovic M, Turna RS, Sanchez-Gonzalez MA, Badger CD, Zorio DA, Singh RK, Wang Y, Jin JP, Ackerman MJ, Pinto JR. In vivo analysis of troponin C knock-in (A8V) mice: evidence that TNNC1 is a hypertrophic cardiomyopathy susceptibility gene. Circ Cardiovasc Genet 8: 653–664, 2015. doi:10.1161/CIRCGENETICS.114.000957. 26304555 PMC4618104

[45] Hwang HS, Kahmini AR, Prascak J, Cejas-Carbonell A, Valera IC, Champion S, Corrigan M, Mumbi F, Parvatiyar MS. Sarcospan deficiency increases oxidative stress and arrhythmias in hearts after acute ischemia-reperfusion injury. Int J Mol Sci 24: 11868, 2023. doi:10.3390/ijms241411868. 37511627 PMC10380899

[46] Hepler C, Shan B, Zhang Q, Henry GH, Shao M, Vishvanath L, Ghaben AL, Mobley AB, Strand D, Hon GC, Gupta RK. Identification of functionally distinct fibro-inflammatory and adipogenic stromal subpopulations in visceral adipose tissue of adult mice. Elife 7: e39636, 2018. doi:10.7554/eLife.39636. 30265241 PMC6167054

[47] Marongiu R. Accelerated ovarian failure as a unique model to study peri-menopause influence on Alzheimer’s disease. Front Aging Neurosci 11: 242, 2019. doi:10.3389/fnagi.2019.00242. 31551757 PMC6743419

[48] Nelson JF, Felicio LS, Randall PK, Sims C, Finch CE. A longitudinal study of estrous cyclicity in aging C57BL/6J mice: I. Cycle frequency, length and vaginal cytology. Biol Reprod 27: 327–339, 1982. doi:10.1095/biolreprod27.2.327. 6889895

[49] Nelson JF, Felicio LS, Osterburg HH, Finch CE. Altered profiles of estradiol and progesterone associated with prolonged estrous cycles and persistent vaginal cornification in aging C57BL/6J mice. Biol Reprod 24: 784–794, 1981. doi:10.1095/biolreprod24.4.784. 7195743

[50] Campbell KP, Lebakken C, Crosbie R, Williamson R. Sarcospan-deficient mouse as a model for clinical disorders associated with sarcospan mutations. PCT/US2000/028035, 1999.

[51] Rossi MA, Basiri ML, McHenry JA, Kosyk O, Otis JM, van den Munkhof HE, Bryois J, Hübel C, Breen G, Guo W, Bulik CM, Sullivan PF, Stuber GD. Obesity remodels activity and transcriptional state of a lateral hypothalamic brake on feeding. Science 364: 1271–1274, 2019. doi:10.1126/science.aax1184. 31249056 PMC7318865

[52] Licholai JA, Nguyen KP, Fobbs WC, Schuster CJ, Ali MA, Kravitz AV. Why do mice overeat high-fat diets? How high-fat diet alters the regulation of daily caloric intake in mice. Obesity (Silver Spring) 26: 1026–1033, 2018. doi:10.1002/oby.22195. 29707908 PMC5970071

[53] Siersbæk MS, Ditzel N, Hejbøl EK, Præstholm SM, Markussen LK, Avolio F, Li L, Lehtonen L, Hansen AK, Schrøder HD, Krych L, Mandrup S, Langhorn L, Bollen P, Grøntved L. C57BL/6J substrain differences in response to high-fat diet intervention. Sci Rep 10: 14052, 2020. doi:10.1038/s41598-020-70765-w. 32820201 PMC7441320

[54] Reynolds TH, Dalton A, Calzini L, Tuluca A, Hoyte D, Ives SJ. The impact of age and sex on body composition and glucose sensitivity in C57BL/6J mice. Physiol Rep 7: e13995, 2019. doi:10.14814/phy2.13995. 30706674 PMC6356156

[55] Matsuzawa Y, Shimomura I, Nakamura T, Keno Y, Kotani K, Tokunaga K. Pathophysiology and pathogenesis of visceral fat obesity. Obes Res 3, *Suppl* 2: 187S–194S, 1995. doi:10.1002/j.1550-8528.1995.tb00462.x. 8581775

[56] Abate N, Garg A, Peshock RM, Stray-Gundersen J, Grundy SM. Relationships of generalized and regional adiposity to insulin sensitivity in men. J Clin Invest 96: 88–98, 1995. doi:10.1172/JCI118083. 7615840 PMC185176

[57] McLaughlin T, Lamendola C, Liu A, Abbasi F. Preferential fat deposition in subcutaneous versus visceral depots is associated with insulin sensitivity. J Clin Endocrinol Metab 96: E1756–E1760, 2011. doi:10.1210/jc.2011-0615. 21865361 PMC3205890

[58] George AM, Jacob AG, Fogelfeld L. Lean diabetes mellitus: an emerging entity in the era of obesity. World J Diabetes 6: 613–620, 2015. doi:10.4239/wjd.v6.i4.613. 25987958 PMC4434081

[59] Liao L, Deng Y, Zhao D. Association of low birth weight and premature birth with the risk of metabolic syndrome: a meta-analysis. Front Pediatr 8: 405, 2020. doi:10.3389/fped.2020.00405. 32850529 PMC7399155

[60] Vaag A, Jensen CB, Poulsen P, Brøns C, Pilgaard K, Grunnet L, Vielwerth S, Alibegovic A. Metabolic aspects of insulin resistance in individuals born small for gestational age. Horm Res 65 Suppl 3: 137–143, 2006. doi:10.1159/000091519. 16612127

[61] Jensen CB, Storgaard H, Dela F, Holst JJ, Madsbad S, Vaag AA. Early differential defects of insulin secretion and action in 19-year-old caucasian men who had low birth weight. Diabetes 51: 1271–1280, 2002. doi:10.2337/diabetes.51.4.1271. 11916955

[62] Wang Y, Rimm EB, Stampfer MJ, Willett WC, Hu FB. Comparison of abdominal adiposity and overall obesity in predicting risk of type 2 diabetes among men. Am J Clin Nutr 81: 555–563, 2005. doi:10.1093/ajcn/81.3.555. 15755822

[63] Crandall DL, Hausman GJ, Kral JG. A review of the microcirculation of adipose tissue: anatomic, metabolic, and angiogenic perspectives. Microcirculation 4: 211–232, 1997. doi:10.3109/10739689709146786. 9219215

[64] Crewe C, An YA, Scherer PE. The ominous triad of adipose tissue dysfunction: inflammation, fibrosis, and impaired angiogenesis. J Clin Invest 127: 74–82, 2017. doi:10.1172/JCI88883. 28045400 PMC5199684

[65] Yi Z, Bishop GA. Regulatory role of CD40 in obesity-induced insulin resistance. Adipocyte 4: 65–69, 2015. doi:10.4161/adip.32214. 26167405 PMC4497298

[66] Nomiyama T, Perez-Tilve D, Ogawa D, Gizard F, Zhao Y, Heywood EB, Jones KL, Kawamori R, Cassis LA, Tschöp MH, Bruemmer D. Osteopontin mediates obesity-induced adipose tissue macrophage infiltration and insulin resistance in mice. J Clin Invest 117: 2877–2888, 2007. doi:10.1172/JCI31986. 17823662 PMC1964510

[67] Hardy OT, Perugini RA, Nicoloro SM, Gallagher-Dorval K, Puri V, Straubhaar J, Czech MP. Body mass index-independent inflammation in omental adipose tissue associated with insulin resistance in morbid obesity. Surg Obes Relat Dis 7: 60–67, 2011. doi:10.1016/j.soard.2010.05.013. 20678967 PMC2980798

[68] Kawai T, Autieri MV, Scalia R. Adipose tissue inflammation and metabolic dysfunction in obesity. Am J Physiol Cell Physiol 320: C375–C391, 2021. doi:10.1152/ajpcell.00379.2020. 33356944 PMC8294624

[69] Weisberg SP, McCann D, Desai M, Rosenbaum M, Leibel RL, Ferrante AW Jr. Obesity is associated with macrophage accumulation in adipose tissue. J Clin Invest 112: 1796–1808, 2003. doi:10.1172/JCI19246. 14679176 PMC296995

[70] Xu H, Barnes GT, Yang Q, Tan G, Yang D, Chou CJ, Sole J, Nichols A, Ross JS, Tartaglia LA, Chen H. Chronic inflammation in fat plays a crucial role in the development of obesity-related insulin resistance. J Clin Invest 112: 1821–1830, 2003. doi:10.1172/JCI19451. 14679177 PMC296998

[71] Klöting N, Fasshauer M, Dietrich A, Kovacs P, Schön MR, Kern M, Stumvoll M, Blüher M. Insulin-sensitive obesity. Am J Physiol Endocrinol Metab 299: E506–E515, 2010. doi:10.1152/ajpendo.00586.2009. 20570822

[72] Wentworth JM, Naselli G, Brown WA, Doyle L, Phipson B, Smyth GK, Wabitsch M, O’Brien PE, Harrison LC. Pro-inflammatory CD11c+CD206+ adipose tissue macrophages are associated with insulin resistance in human obesity. Diabetes 59: 1648–1656, 2010. doi:10.2337/db09-0287. 20357360 PMC2889764

[73] Tessaro FH, Ayala TS, Nolasco EL, Bella LM, Martins JO. Insulin influences LPS-induced TNF-alpha and IL-6 release through distinct pathways in mouse macrophages from different compartments. Cell Physiol Biochem 42: 2093–2104, 2017. doi:10.1159/000479904. 28810254

[74] Ballak DB, Stienstra R, Tack CJ, Dinarello CA, van Diepen JA. IL-1 family members in the pathogenesis and treatment of metabolic disease: focus on adipose tissue inflammation and insulin resistance. Cytokine 75: 280–290, 2015. doi:10.1016/j.cyto.2015.05.005. 26194067 PMC4553099

[75] Hannum CH, Wilcox CJ, Arend WP, Joslin FG, Dripps DJ, Heimdal PL, Armes LG, Sommer A, Eisenberg SP, Thompson RC. Interleukin-1 receptor antagonist activity of a human interleukin-1 inhibitor. Nature 343: 336–340, 1990. doi:10.1038/343336a0. 2137200

[76] Pyrillou K, Burzynski LC, Clarke MC. Alternative pathways of IL-1 activation, and its role in health and disease. Front Immunol 11: 613170, 2020. doi:10.3389/fimmu.2020.613170. 33391283 PMC7775495

[77] Mills KH. IL-17 and IL-17-producing cells in protection versus pathology. Nat Rev Immunol 23: 38–54, 2023. doi:10.1038/s41577-022-00746-9. 35790881 PMC9255545

[78] Zúñiga LA, Shen WJ, Joyce-Shaikh B, Pyatnova EA, Richards AG, Thom C, Andrade SM, Cua DJ, Kraemer FB, Butcher EC. IL-17 regulates adipogenesis, glucose homeostasis, and obesity. J Immunol 185: 6947–6959, 2010 [Erratum in *J Immunol* 186: 1291, 2011]. doi:10.4049/jimmunol.1001269. 21037091 PMC3001125

[79] Brainard RE, Watson LJ, Demartino AM, Brittian KR, Readnower RD, Boakye AA, Zhang D, Hoetker JD, Bhatnagar A, Baba SP, Jones SP. High fat feeding in mice is insufficient to induce cardiac dysfunction and does not exacerbate heart failure. PloS One 8: e83174, 2013 [Erratum in *PLoS One* 9: e113944, 2014]. doi:10.1371/journal.pone.0083174. 24367585 PMC3867436

[80] Tadinada SM, Weatherford ET, Collins GV, Bhardwaj G, Cochran J, Kutschke W, Zimmerman K, Bosko A, O’Neill BT, Weiss RM, Abel ED. Functional resilience of C57BL/6J mouse heart to dietary fat overload. Am J Physiol Heart Circ Physiol 321: H850–H864, 2021. doi:10.1152/ajpheart.00419.2021. 34477461 PMC8616610

[81] Nayor M, Enserro DM, Xanthakis V, Larson MG, Benjamin EJ, Aragam J, Mitchell GF, Vasan RS. Comorbidities and cardiometabolic disease: relationship with longitudinal changes in diastolic function. JACC Heart Fail 6: 317–325, 2018. doi:10.1016/j.jchf.2017.12.018. 29525334 PMC5878123

[82] Murphy E, Amanakis G, Fillmore N, Parks RJ, Sun J. Sex differences in metabolic cardiomyopathy. Cardiovasc Res 113: 370–377, 2017. doi:10.1093/cvr/cvx008. 28158412 PMC5852638

[83] Erkens R, Kramer CM, Lúckstädt W, Panknin C, Krause L, Weidenbach M, Dirzka J, Krenz T, Mergia E, Suvorava T, Kelm M, Cortese-Krott MM. Left ventricular diastolic dysfunction in Nrf2 knock out mice is associated with cardiac hypertrophy, decreased expression of SERCA2a, and preserved endothelial function. Free Radic Biol Med 89: 906–917, 2015. doi:10.1016/j.freeradbiomed.2015.10.409. 26475037

[84] Murphy SP, Kakkar R, McCarthy CP, Januzzi J, Jr. Inflammation in heart failure: JACC State-of-the-Art Review. J Am Coll Cardiol 75: 1324–1340, 2020. doi:10.1016/j.jacc.2020.01.014. 32192660

